# A 200-year annually laminated stalagmite record of precipitation seasonality in southeastern China and its linkages to ENSO and PDO

**DOI:** 10.1038/s41598-018-30112-6

**Published:** 2018-08-17

**Authors:** Haiwei Zhang, Hai Cheng, Christoph Spötl, Yanjun Cai, Ashish Sinha, Liangcheng Tan, Liang Yi, Hong Yan, Gayatri Kathayat, Youfeng Ning, Xianglei Li, Fan Zhang, Jingyao Zhao, R. Lawrence Edwards

**Affiliations:** 1 Institute of Global Environmental Change, Xi’an Jiaotong University, Xi’an, 710054 China; 2 Institute of Earth Environment, Chinese Academy of Sciences, State Key Laboratory of Loess and Quaternary Geology, Xi’an, 710061 China; 3 Institute of Geology, University of Innsbruck, Innsbruck, 6020 Austria; 4 Department of Earth Science, University of Minnesota, Minneapolis, Minnesota 55455 USA; 5 Department of Earth Science, California State University Dominguez Hills, Carson, California 90747 USA; 6 State Key Laboratory of Marine Geology, Tongji University, Shanghai, 200092 China

## Abstract

In southeastern China (SEC), the precipitation amount produced by the East Asian summer monsoon (EASM) is almost equivalent to that during the non-summer monsoon (NSM) period, both of them significantly affecting agriculture and socioeconomy. Here, we present a seasonally-resolved stalagmite δ^18^O record (δ^18^O_s_) for the interval 1810–2009 AD from E’mei cave, Jiangxi Province, SEC. The comparison between δ^18^O_s_ and instrumental data indicates that the δ^18^O_s_ variability is primarily controlled by the precipitation seasonality (i.e., the ratio of EASM/NSM precipitation) modulated by the El Niño/Southern Oscillation (ENSO) on interannual to interdecadal timescales. Higher (lower) δ^18^O_s_ values thereby correspond to lower (higher) EASM/NSM ratios associated with El Niño (La Niña) events. Significant correlations with ENSO and the Pacific Decadal Oscillation (PDO) indicate that the precipitation seasonality in SEC is remarkably influenced by ocean-atmosphere interactions, with lower (higher) EASM/NSM ratios during warm (cold) phases of ENSO/PDO. The progressive increase in δ^18^O_s_ since 2005 AD may reflect a strengthening of the central Pacific El Niño under continued anthropogenic global warming. The relationship between seasonal precipitation and δ^18^O_s_ with ENSO/PDO requires further studies.

## Introduction

Southeastern China (SEC), which is one of the most densely populated areas in East Asia, is significantly influenced by summer floods and droughts associated with the East Asian summer monsoon (EASM)^1^. For example, in 1998, more than 1,000 people were killed and more than 200 million people were affected by the floods in the middle and lower reaches of Yangtze River Valley (YRV), and the direct socioeconomic losses amounted to about £10,000 million^2^. In addition, the spring rainy period in SEC^3^, associated with low temperatures, overcast and rain, is also a severe threat to agriculture (e.g., cotton plants) and transportation^4,5^. Studies indicate that the rainy season in SEC includes the EASM and spring rainfall and both of them show high variability^4,6^. Frequent flood and drought events resulting from this seasonally variable precipitation seriously impact agriculture and socioeconomy in this region. Therefore, it is essential to study the variability in precipitation seasonality and assess its influencing factors.

Observations and simulations demonstrate that ENSO and PDO have a major impact on the interannual-interdecadal variability in precipitation over SEC^7–17^. Some investigated the combined effects of PDO^7–9^ and ENSO^10–15^ on EASM, spring or East Asian winter monsoon (EAWM) precipitation over Eastern China, respectively. Others examined the modulation of the relationship between ENSO and EASM^11,16^, spring^6^ or EAWM^17^ precipitation by the PDO. However, the relationship between precipitation variability and ENSO/PDO on interannual to interdecadal timescales remains poorly understood, partly due to the limited availability of instrumental datasets. In addition, most researchers focused on the variations of EASM or EAWM in the Asian monsoon region, and only few studies examined the variability in precipitation seasonality^18^. Long-term paleoclimatic reconstructions are needed to explore the variability of precipitation in SEC and its seasonality.

Some δ^18^O records of tree-ring cellulose in SEC were recently published^18–20^, but they mainly constrain precipitation and relative humidity during the rainy season. High-precision and high-resolution δ^18^O records of stalagmite (δ^18^O_s_), calibrated against instrumental data, can be used to reconstruct the variability of ENSO/PDO in the past. In this study, we present a first seasonally-resolved δ^18^O_s_ record for the period 1810–2009 AD based on an annually laminated stalagmite from E’mei cave, Jiangxi Province, SEC. This record allows us to explore the variability in the precipitation seasonality modulated by ENSO and PDO on interannual to interdecadal timescales.

## Cave Location, Sample and Climate

**Figure 1:**
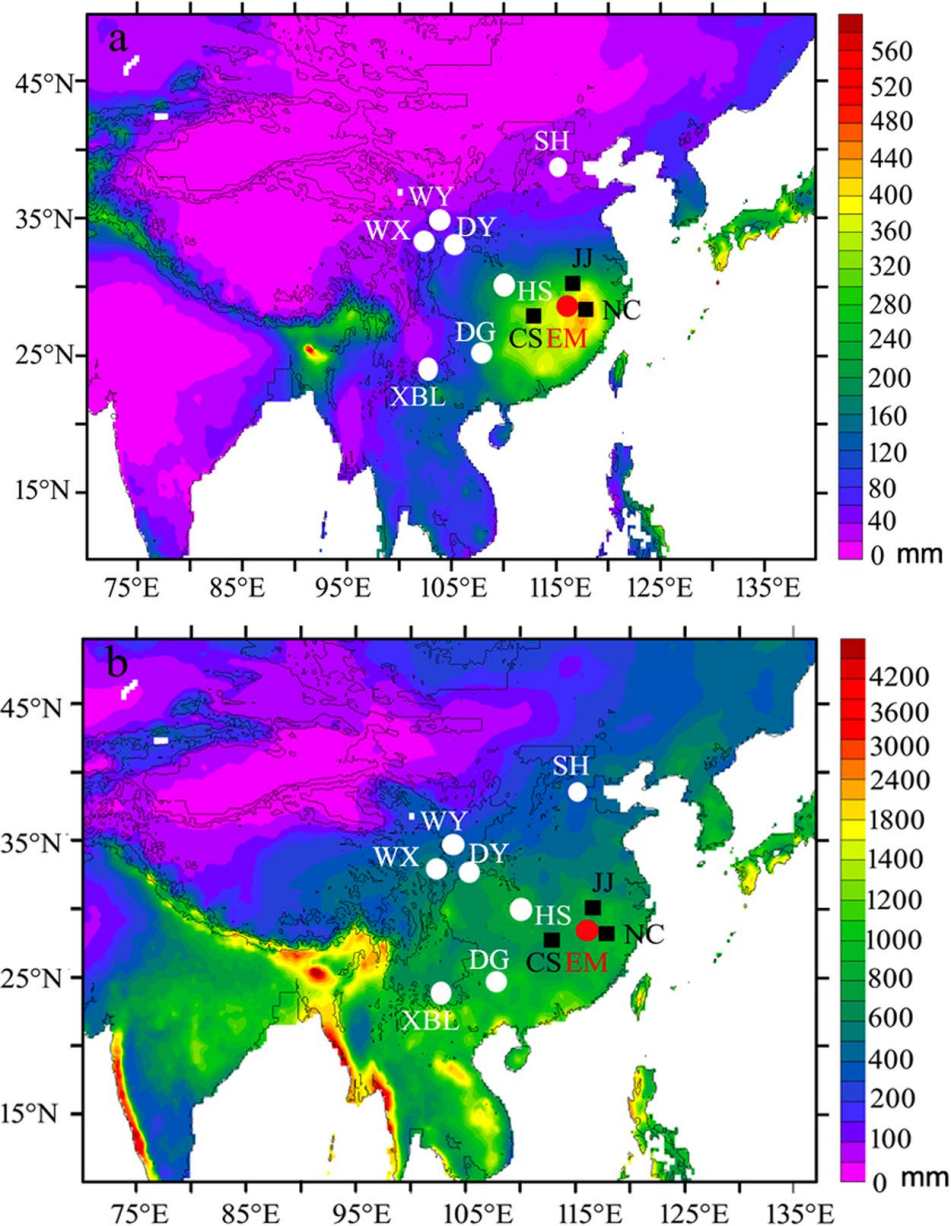
Overview map showing the spatial distribution of seasonal precipitation amount in China and other locations mentioned in this study. The backgrounds in (**a**) and **(b**) are the distribution of mean SPR (March–April) and EASM (May–September) precipitation amount in China from 1951 to 2007, respectively. The red dot denotes the location of E’mei Cave (this study), and the white dots indicate the Shihua^28^ (SH), Wuya^22^ (WY), Wanxiang^27^ (WX), Dayu^47^ (DY), Heshang^61^ (HS), Dongge^38^ (DG) and Xiaobailong^62^ (XBL) cave sites in the monsoon region of China. The black squares mark the Jiujiang (JJ), Nanchang (NC) and Changsha (CS) meteorological stations. The map was exported based on the precipitation data source (1951–2007) from APHRODITE (Asian Precipitation-Highly-Resolved Observational Data Integration Towards Evaluation of Water Resources, APHRO_MA_V1101R2 product, (21). Website: http://www.chikyu.ac.jp/precip/)^63^.

E’mei Cave (115°29′44″E, 29°33′18″N, 53 m a.s.l.) is located in the north of Jiangxi Province, SEC (Fig. 1). The hostrock is a Middle Ordovician limestone. An actively growing calcitic stalagmite (EM1) of 60 mm in height (Supplementary Fig. S1) was collected ~150 m behind the entrance of the cave in December 2009. The regional climate is subtropical humid and the rainy season includes spring (March to May) and summer (June to August) (Supplementary Fig. S2). Instrumental data from the Nanchang and Jiujiang meteorological stations (1951–2010 AD) show that mean annual precipitation is 1514 mm and the annual temperature is 17.3 °C. The EASM (May to September) precipitation with lower δ^18^O values accounts for 54% of the annual precipitation and the non-summer monsoon (NSM) (October to next April) precipitation with higher δ^18^O values accounts for 46% (Supplementary Fig. S2). The amount-weighted mean annual precipitation δ^18^O (δ^18^O_w_) value is controlled by both EASM and NSM precipitation. Data from the nearest GNIP station (Changsha station, about 100 km west of E’mei Cave) during 1988–1992 indicate lower δ^18^O_w_ values, more June-September precipitation and less March-April precipitation during La Niña years as compared to El Niño years (Supplementary Fig. S3). Therefore, the variation in δ^18^O_w_ during 1988–1992 is probably influenced by the ratio of EASM/NSM precipitation but not by the amount of annual precipitation which lacks a significant variation (Supplementary Fig. S3).

## Results

### Chronology

Elevated detrital ^232^Th levels of 2–4 × 10^3^ ppt resulted in large dating uncertainties of 172–438 years for ^230^Th dates (Supplementary Table S1 and Fig. S4) and hinder precise age models. Although the dating errors are large, the ^230^Th dates still indicate that stalagmite EM1 grew during about last 200 years, which is consistent with the 200 ± 3 well-developed laminae within errors (see next paragraph). The ^230^Th dates at 6 and 18.5 mm from the top are 1997 ± 267 and 1932 ± 158 AD, respectively, and the inferred age of the top of EM1 is 2028 ± 267 AD. This is consistent with the observation of active dripping when this stalagmite was collected in December 2009. The top age of EM1 was therefore assumed to be late 2009 AD.

The cross-section of EM1 shows continuous, well-developed laminae consisting of alternating dense, translucent sub-layers (TDSL) and white, porous sub-layers (WPSL)^21,22^ (Supplementary Fig. S1). The visible WPSL are opaque under the transmitted-light microscope, but brightly luminescent under mercury light source UV reflected light and in the confocal laser fluorescent microscope (CLFM) (Supplementary Fig. S1), which is consistent with observations from other caves^21,23^. As suggested by previous studies, the fluorescent sub-layer forms from organic-rich dripwater during the wet season, while dripwater depleted in organics during the dry season results in the non-fluorescent sub-layer^23^. We identified a total of 159 δ^18^O_s_ cycles between 0 and 45.6 mm from the top of EM1, consistent with 159 bright-dark couplets, confirming the annual nature of these highly regular laminae (Supplementary Fig. S5b and c). 200 ± 3 bright-dark couplets were counted using CLFM between 0 and 53.6 mm from the top of EM1. The number of annual laminae and δ^18^O_s_ cycles was used to establish an age model for EM1 (Supplementary Figs S4 and S5), considering that EM1 grew continuously from 1810 to 2009 AD, which is consistent with the ^230^Th dates within errors.

In order to constrain the chronology of EM1, we also compared the δ^18^O and δ^13^C records of EM1 with stalagmite YQ15-1, which was obtained from Yongquan Cave, 50 km northeast of E’mei Cave. The dating errors of YQ15-1 (12–15 years, Supplementary Fig. S5a) are much smaller than those of EM1. The δ^18^O and δ^13^C records of YQ15-1 and EM-1 replicate well (Supplementary Fig. S5a), further confirming the EM1 chronology.

### δ^18^O_s_ record

**Figure 2:**
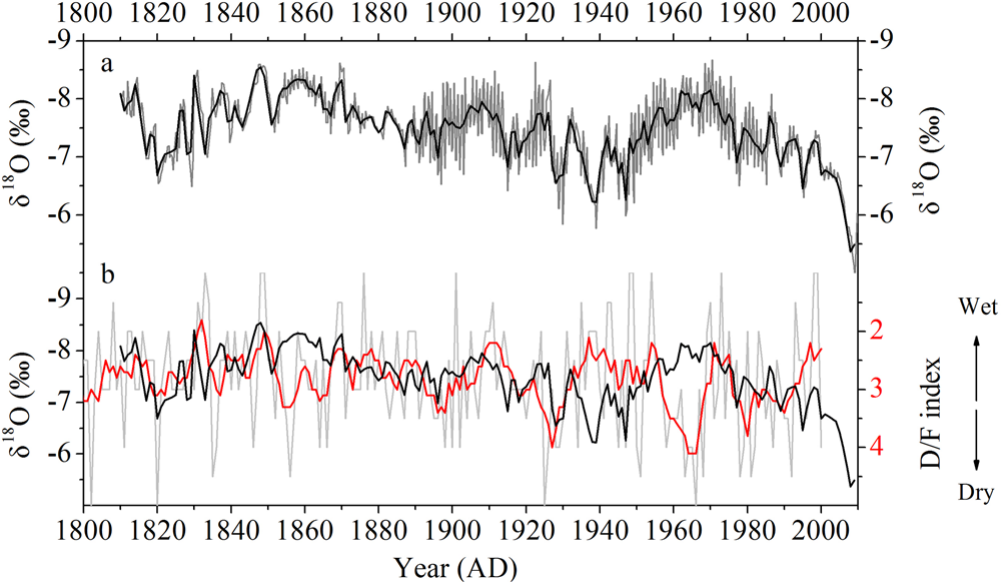
(**a**) The original δ^18^O (grey line) record and the averaged annually resolved δ^18^O (black line) record of stalagmite EM1. (**b**) Comparison between annually resolved EM1 δ^18^O (black line) and D/F index (hoar line) records. The red line is the 5-year smoothing average curve of D/F index. The annual D/F index, based on the abundance of drought and flood events during spring, summer and autumn as recorded in historical documents^39^ reflects the local precipitation variation.

There are clear annual δ^18^O_s_ cycles between 0 and 45.6 mm from the top of EM1, while these cycles are less clear between 45.6 and 53.6 mm because of a slower growth rate (Supplementary Fig. S4). We therefore interpolated this interval by using the corresponding relationship between the micromilling track of stable isotope analyses and layer counting (Supplementary Fig. S1b). Together with the 159 annual δ^18^O_s_ cycles between 0 and 45.6 mm, δ^18^O and δ^13^C records of EM1 were established for the period 1810–2009 AD (Fig. 2a). The good replication of the stable isotope records between EM1 and YQ15-1 indicates that EM1 was deposited close to isotope equilibrium conditions^24,25^, although “Hendy tests”^26^ yielded a significant correlation (r = 0.31, p < 0.01, n = 780) between δ^18^O and δ^13^C values along the growth axis. The co-variation of δ^18^O and δ^13^C can also result from the climatic and environmental change^24^. The inverse relationship between δ^18^O and δ^13^C values on seasonal timescales (Supplementary Fig. S5b) further indicates that kinetic isotope fractionation has a negligible effect only and the δ^18^O_s_ signal recorded in EM1 is primarily of climatic origin.

## Discussion

### Significance of δ^18^O_s_ proxy

Although δ^18^O_s_ variability in the monsoon region of China on orbital to millennial timescales generally reflects the variation of EASM intensity, its significance on short timescales remains to be understood. Previous studies demonstrated that the δ^18^O_s_ variability in the marginal zone of the EASM reflects the variation of monsoon intensity or precipitation amount^22,27,28^. In SEC, however, this relationship is more complex, because of the various factors influencing precipitation δ^18^O (δ^18^O_p_), e.g., precipitation amount, moisture sources, moisture pathways and rainfall seasonality. Dayem *et al*.^29^ suggested that several processes associated with precipitation seasonality might jointly influence the fluctuation of Chinese δ^18^O_s_^29^. Based on the analyses of modern precipitation δ^18^O data^30^, Tan^31^ suggested that δ^18^O_s_ in the monsoon region of China records the moisture source changes related to ENSO circulation but not the monsoon intensity or precipitation amount. The higher δ^18^O_s_ values reflect more moisture originating from the adjacent Pacific Ocean relative to the remote Indian Ocean during El Niño phases, and vice versa^31^. However, some studies suggested that EASM precipitation is primarily derived from the Indian Ocean, while the moisture from the West Pacific Ocean is a minor contributor to monsoonal rainfall only^32,33^, and the δ^18^O_p_ variability is caused by both precipitation amount and moisture transport history^32^. Cheng *et al*.^34^ and Yang *et al*.^35^ suggested that δ^18^O_s_ is primarily controlled by large-scale monsoon intensity and upstream precipitation. As introduced above, δ^18^O_w_ in the study area is influenced by both the EASM and the NSM associated with ENSO circulation (Supplementary Fig. S2). Thus, the EM1 δ^18^O_s_ record was compared to the instrumental seasonal precipitation and the Southern Oscillation Index (SOI) in order to explore its significance on interannual to interdecadal timescales.

**Figure 3:**
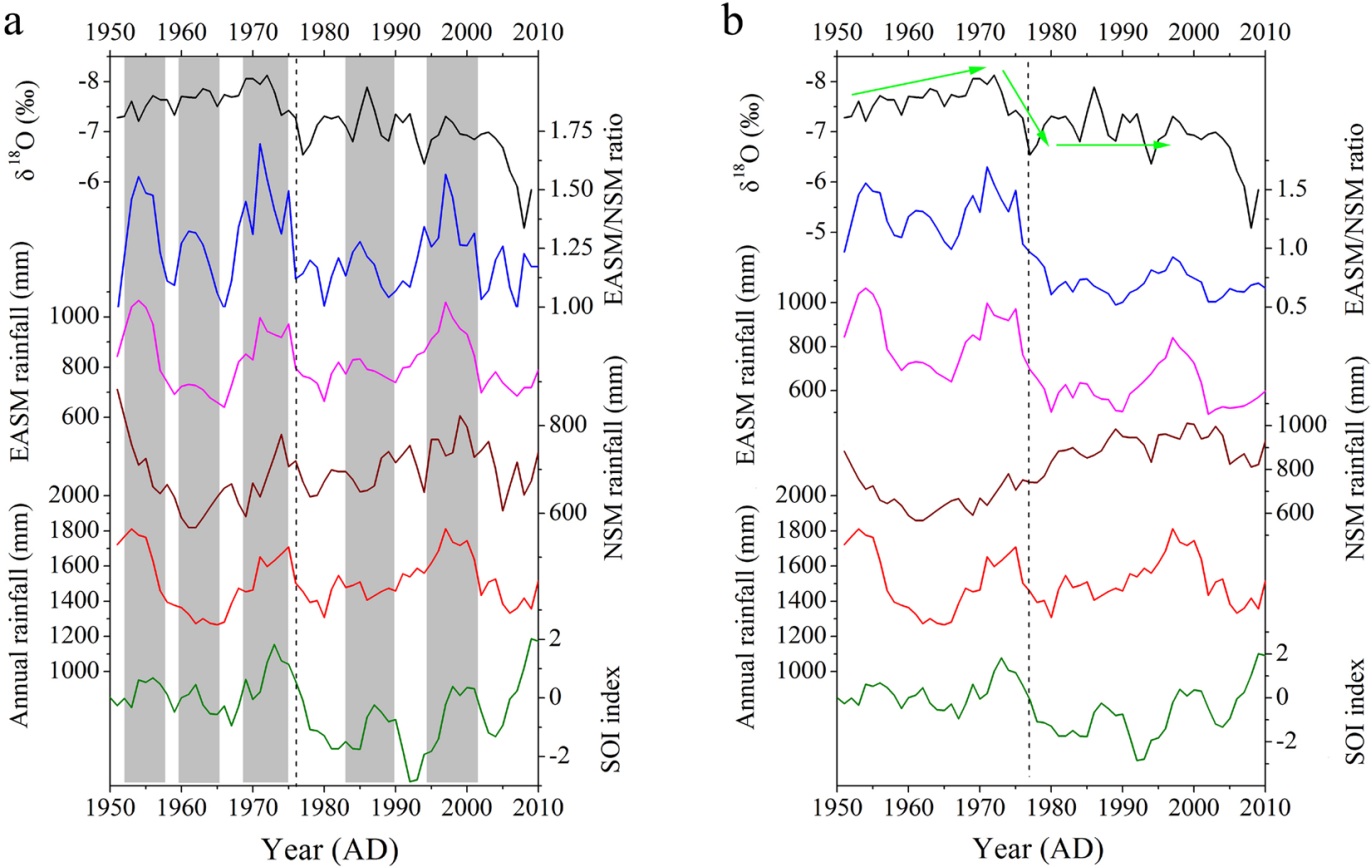
Comparison between EM1 δ^18^O (black line), SOI index (green line) and instrumental precipitation (EASM (pink), NSM (brown) and annual (red) precipitation and EASM/NSM ratio (blue)) in the study area during 1951–2010 AD. In (**a**) the EASM rainfall is represented by May-September precipitation and NSM rainfall is represented by October-April precipitation during 1951–2010 AD; In (**b**) the May-September and October-April precipitation is used to represent EASM and NSM precipitation in 1951–1976 AD, respectively. The June-September and October-May precipitation is used to represent EASM and NSM precipitation for 1977–2010 AD, respectively. EASM and NSM precipitation was adjusted after the shifts of PDO/ENSO in 1976–1977 AD (dashed line), because the EASM starts late (early) and tends to be weaker (stronger) during El Niño (La Niña) years^41^.

On an interannual timescale, the comparison between δ^18^O_s_ and the SOI shows a significantly negative correlation (Fig. 3a, r = −0.50, p < 0.01 using a 5-year moving average for 1951–2004 AD), with higher (lower) δ^18^O_s_ during El Niño (La Niña) events. This is consistent with previous studies based on instrumental data^30,33,35^ and δ^18^O records of tree-ring cellulose^18–20,36^ and stalagmites^37,38^ in SEC. δ^18^O_s_ is significantly and negatively correlated with the ratio of EASM/NSM precipitation (r = −0.32, p < 0.05) using a 5-year moving average for 1951–2009 AD (Fig. 3a). There is no significant correlation between δ^18^O_s_ and the EASM, NSM and annual precipitation, although a good coherent variation can be observed (grey bars in Fig. 3a). We also compared the interannual variation between EM1 δ^18^O_s_ and the local Drought/Flood (D/F) index for 1810–2000 AD. The annual D/F index, based on drought or flood events in spring, summer and autumn recorded in historical documentaries^39^, can reflect local precipitation variations in May-September, with a decreased (increased) D/F index representing more (less) precipitation. The good coherence indicates that lower (higher) δ^18^O_s_ values are associated with increased (decreased) EASM precipitation in the study area (Fig. 2b).

PDO and ENSO are strongly correlated on decadal timescales^11^, with warm PDO phases (positive PDO index) corresponding to warm ENSO phases (El Niño phases, negative SOI index). On an interdecadal timescale, previous studies demonstrated that the PDO/ENSO circulation shifted from a cold phase during 1951–1976 AD to a warm phase during 1977–2000 AD^7,11^. During the cold phase of PDO/ENSO, EM1 δ^18^O_s_ values are generally lower than during the subsequent warm phase (green arrows in Fig. 3a), whereas precipitation amount does not show a significantly systematic shift (Fig. 3a). Actually, studies demonstrated that the intensity and duration of the EASM have a strong variability on interdecadal timescales^40^. The EASM starts late (early) and tends to be weaker (stronger) during El Niño (La Niña) years^41^, and the onset varies from late April to early June^40,41^. Thus, May-September precipitation cannot be always used to represent the EASM precipitation, especially in SEC characterized by a strong precipitation seasonality. This is also supported by instrumental δ^18^O_p_ data from the Changsha GNIP station (Supplementary Fig. S6). They show that the δ^18^O_p_ values in May during La Niña years (1988–1989 AD) are lower than those during El Niño years (1991–1992 AD), indicating that the EASM starts early (late) during La Niña events (El Niño events). Therefore, EASM precipitation during 1951–1976 AD likely represented May-September precipitation, while it shifted to June-September precipitation during 1977–2010 AD; the NSM precipitation should be adjusted accordingly. Hence, less EASM and more NSM precipitation results in higher δ^18^O_s_ values during long-term El Niño phases (Fig. 3b) and vice versa.

Similarly, the poor correlation between δ^18^O_s_ and the D/F index during 1930–1970 AD on an interdecadal timescale (Fig. 2b) might be caused by the variation of precipitation seasonality associated with shifts in oceanic-atmospheric circulation. We calculated the correlation coefficients between δ^18^O_s_ and the adjusted EASM and NSM precipitation as well as EASM/NSM ratios using a 5-year moving average for 1951–2009 AD. The data show that δ^18^O_s_ is significantly negatively correlated with the ratio of EASM/NSM precipitation (r = −0.67, p < 0.01) and the EASM precipitation (r = −0.54, p < 0.01), and is significantly positively correlated with the NSM precipitation (r = 0.58, p < 0.01), suggesting that higher (lower) δ^18^O_s_ values are associated with lower (higher) EASM/NSM ratios during El Niño (La Niña) phases on interannual to interdecadal timescales.

Considering the strong precipitation seasonality (including precipitation amount and moisture sources/pathways) in the study area, we suggest that the EASM/NSM ratio better explains the δ^18^O_s_ variation on interannual-interdecadal timescales. Studies demonstrated that EASM precipitation is mainly sourced from the remote Indian Ocean while NSM precipitation primarily originates from the adjacent Pacific Ocean^32,33^. Thus, the EASM/NSM ratio may also represent the ratio of moisture sources/pathways during the EASM season to that of the NSM season, partly similar to the “circulation effect” proposed by Tan^31^. He suggested that the δ^18^O_p_ and δ^18^O_s_ values are affected by the variation of moisture source in the summer monsoon season. We also emphasize the influence of NSM precipitation, which accounts for 45~65% of the annual precipitation in the study area. As a consequence, we suggest that the variation in precipitation seasonality (e.g., EASM/NSM ratio) modulated by ENSO plays a key factor in controlling the δ^18^O_s_ variability in the study area on interannual to interdecadal timescales, with higher (lower) δ^18^O_s_ values reflecting lower (higher) EASM/NSM ratios associated with El Niño (La Niña) phases.

### Links between δ^18^O_s_, precipitation seasonality and ocean-atmosphere interaction

**Figure 4:**
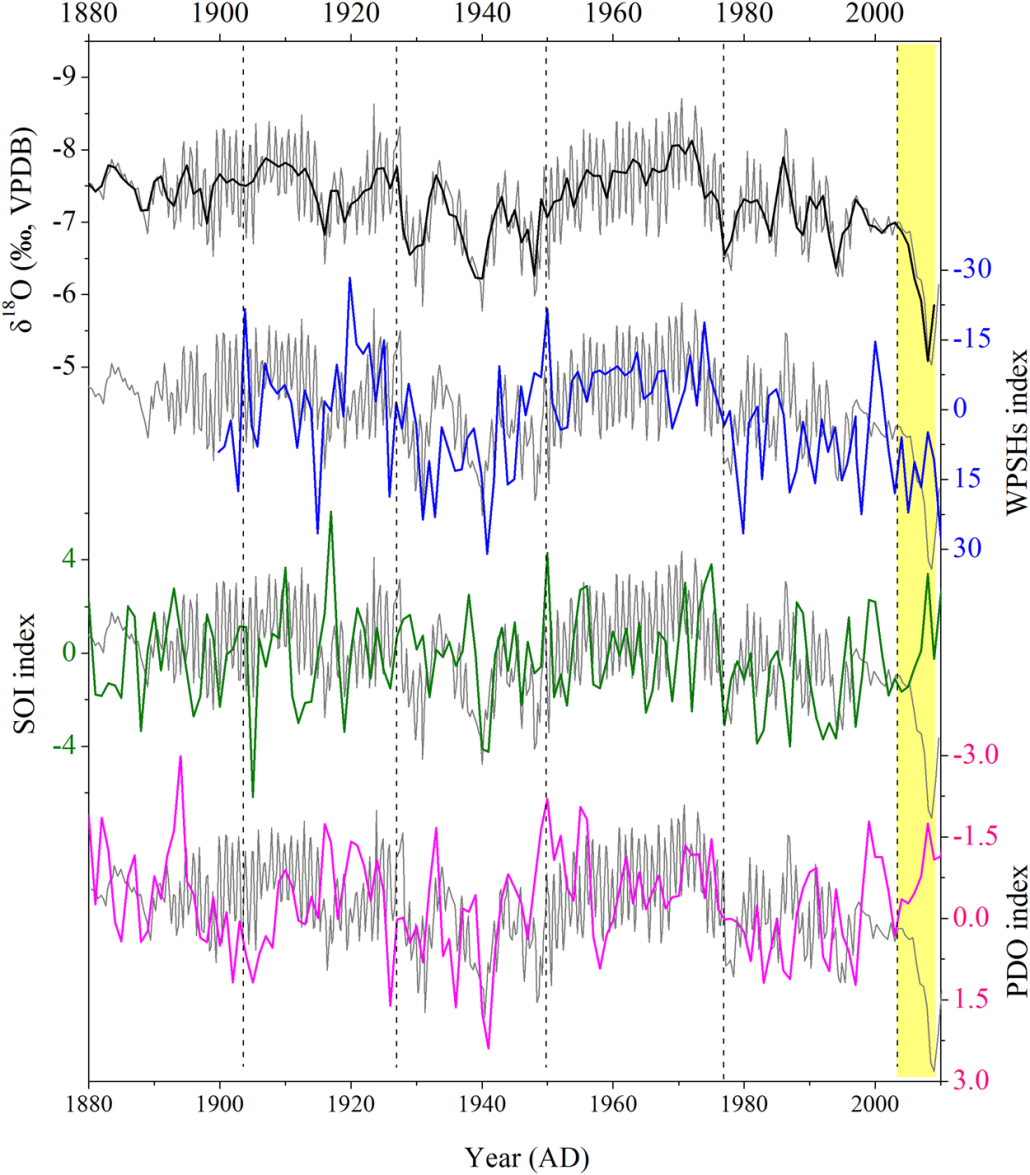
Comparison between annually resolved EM1 δ^18^O (black), WPSH_s_ (blue), SOI (green) and PDO (pink) records during 1880–2009 AD. EM1 δ^18^O is positively correlated with the PDO (r = 0.32, p < 0.01) and negatively correlated with the SOI index (r = −0.27, p < 0.01) after applying a 5-year average for 1880–2004 AD. The grey lines indicate the original EM1 δ^18^O record. The progressively increasing EM1 δ^18^O values since 2005 AD (yellow bar) interrupt the coherent correlation with ENSO and PDO. A significantly positive correlation (r = 0.61, p < 0.01) between δ^18^O and WPSH_s_ is observed after applying a 5-year moving average for 1900–2009 AD.

The comparison between EM1 δ^18^O_s_ and the PDO index shows a significantly positive correlation (Fig. 4, r = 0.27, p < 0.01, applying a 5-year average for 1854–2004 AD), with higher (lower) δ^18^O_s_ values corresponding to warm (cold) PDO phases. A positive correlation between the PDO and the δ^18^O record was also observed in other records from speleothem^22,28,42–45^ and tree-ring cellulose^36,46^ in the monsoon region of China. The EM1 δ^18^O_s_ is significantly negatively correlated with the SOI index (Fig. 4, r = −0.32, p < 0.01, applying a 5-year average for 1866–2004 AD), with higher (lower) δ^18^O_s_ values corresponding to El Niño (La Niña) phases. This is consistent with the relationship between δ^18^O_s_ and the SOI index during 1951–2004 AD (Fig. 3) and other studies based on simulations and reconstructions discussed above. Studies demonstrated that the effect of ENSO or PDO on EASM precipitation in SEC is significant by modulating the position and intensity of the western Pacific subtropical high (WPSH), which changes under different phases of PDO/ENSO^1,8,40^. WPSH intensities enhance (weaken) and the western ridge point moves westward (eastward) during El Niño (La Niña) phases^41^. Based on a comparison between instrumental δ^18^O_p_ data and the WPSH strength (WPSH_s_) index, Tan^30^ showed that increased δ^18^O_p_ values in SEC are associated with a strengthening and westward expansion of the WPSH during the summer following an El Niño event. The comparison between the WPSH_s_ index and δ^18^O_s_ yielded a significantly positive correlation (Fig. 4, r = 0.61, p < 0.01, after applying a 5-year moving average for 1900–2009 AD), with higher (lower) δ^18^O_s_ corresponding to a strengthened (weakened) WPSH. Positive correlations between WPSH intensity and δ^18^O records were also reported from the monsoon region of China based on stalagmites and tree-ring cellulose^19,22,31^. As a consequence, the comparison between δ^18^O_s_, WPSH_s_, ENSO and PDO reveals that higher (lower) δ^18^O_s_ values represent a strengthened (weakened) WPSH associated with warm (cold) phases of PDO/ENSO on interannual to interdecadal timescales. This indicates a lower (higher) EASM/NSM ratio in the study area during a warm (cold) phase of PDO/ENSO. The significant 55–66 year cycle observed in the EM1 δ^18^O_s_ time series is generally consistent with the 50–70 year cycle seen in the PDO records^22^. The significant 2.4, 3.2 and 5.7-year cycles in the EM1 δ^18^O_s_ time series are consistent with 2–7 year cycles in ENSO records, further supporting the important influence of PDO and ENSO on precipitation variation (Supplementary Fig. S7).

**Figure 5:**
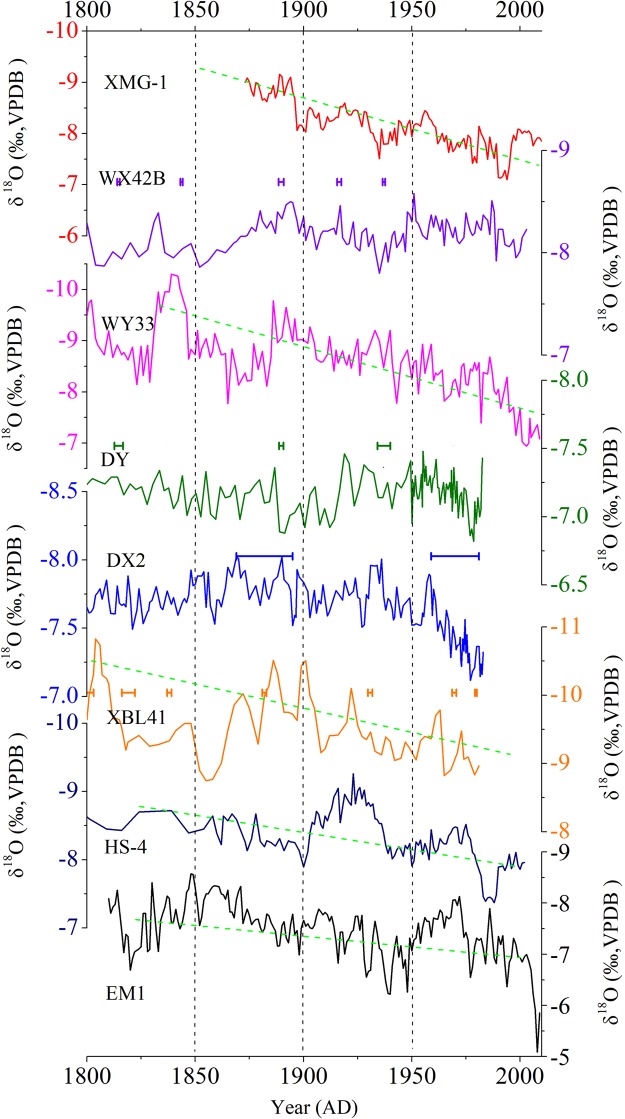
Comparison between high-resolution δ^18^O records from annually laminated and high-precisely ^230^Th dated stalagmites in the monsoon region of China: Shihua Cave^28^ (red), Wangxiang Cave^27^ (purple), Wuya Cave^22^ (pink), Heshang Cave^61^ (dark blue), Dayu Cave^47^ (green), Dongge Cave^38^ (blue), Xiaobailong Cave^62^ (orange) and E’mei Cave (black, this study). The error bars of the records from Wanxiang, Dayu, Xiaobailong and Dongge caves are also shown. There is no error bar for the other records, because their chronologies are based on the annual layer counting combined with ^230^Th dates (with larger dating errors).

A comparison of available high-resolution δ^18^O_s_ records from annually laminated or high-precisely ^230^Th-dated stalagmites in the monsoon region of China shows a consistent long-term increasing trend (Fig. 5), indicating a gradual weakening of the EASM intensity associated with the large-scale weakening of the Asian summer monsoon^28^. However, these δ^18^O_s_ records show differences in long-term trends and amplitude. For example, δ^18^O_s_ records from Wanxiang, Dayu and Dongge caves do not show the long-term increasing trend during last 200 years, and their interdecadal amplitudes are smaller than in other records (Fig. 5). The dating errors of the records from Wanxiang, Dayu, Xiaobailong and Dongge caves are less than 5 years, except for Dongge record. The records from Shihua, Wuya, Heshang and E’mei caves are based on annual layer counting combined with ^230^Th dates. Therefore, these differences should not result from the chronology uncertainties. If δ^18^O_s_ is influenced by the “circulation effect”, δ^18^O_s_ in SEC, a region more sensitive to circulation changes, should show larger amplitudes. Actually, the long-term δ^18^O_s_ amplitudes of stalagmites from Heshang, Dongge and E’mei caves are smaller than those from Shihua and Wuya caves in North China and Xiaobailong cave in Northwest China (green dotted lines in Fig. 5). In addition, the interdecadal variations are not coherent in these δ^18^O_s_ records from the monsoon region of China, and similarly inconsistent changes are also seen in other studies on decadal-centennial timescales^25,47^. This further demonstrates that δ^18^O_s_ is not only affected by circulation but also by precipitation amount, because the migration of the EASM rainbelt results in different precipitation patterns in the monsoon region of China. Except for the variations in EASM precipitation, changes in NSM precipitation also contribute to the interdecadal divergences seen in the EM1 δ^18^O_s_ record.

What is the relationship between δ^18^O_s_ and seasonal precipitation amount in the study area during different PDO/ENSO phases? On an interannual timescale lower δ^18^O_s_ values are associated with more EASM precipitation during La Niña phases (Figs 2b and 3a) and vice versa. By analyzing the precipitation variability in East China during 14 El Niño events since 1950s Kong and Tu^48^ found that less EASM precipitation in May-September in the lower reaches of YRV are associated with El Niño events. Xu *et al*.^20^ showed that the interannual variability in tree-ring cellulose δ^18^O data in SEC reveals the variability in May-October precipitation or relative humidity associated with ENSO, with higher (lower) δ^18^O values corresponding to less (more) precipitation during El Niño (La Niña) events. Studies based on instrumental and reanalysis data suggest increased spring^5,13^ and EAWM precipitation^12,14,15^ in SEC associated with El Niño events, indicating increased NSM precipitation during El Niño events on interannual timescales. Therefore, we suggest that the interannual variability recorded by the EM1 δ^18^O_s_ data might also reflect the variability in seasonal precipitation amount, with more (less) EASM and less (more) NSM precipitation during La Niña (El Niño) phases.

On interdecadal timescales, many studies demonstrate that more summer monsoon precipitation in June-August in the YRV region results from warm PDO/ENSO phases, and vice versa^7,8,49,50^. These studies indicate that decreased summer rainfall in North China and increased summer rainfall in South China (called “north drought-south flood”)^49^ result from a strong EASM during cold phases of PDO/ENSO, and vice versa. However, Chan and Zhou^11^ found that less (more) early summer monsoon precipitation in May-June in South China results from the warm (cold) phases of PDO/ENSO. This indicates that the pattern of interdecadal variability of June-August precipitation is opposite to that of May-June precipitation associated with PDO/ENSO. Yang *et al*.^9^ also demonstrated that the effect of the PDO on precipitation anomalies in East China is depends on the month: May-June precipitation patterns in East China are opposite July-August or annual mean patterns. In the study area, the interdecadal variation of precipitation in May also shows an opposite trend to that in June-September for 1951–2009 AD (Supplementary Fig. S8). This further supports our hypothesis that either May-September or June-August precipitation should be used to represent EASM precipitation under different phases of PDO/ENSO (Fig. 3b). Recent observations and simulations indicate that increased (decreased) spring^6^ and EAWM^17^ precipitation in SEC occurred during warm (cold) phases of ENSO/PDO on interdecadal timescales, indicating more (less) NSM precipitation during warm (cold) phases of ENSO/PDO. This is consistent with our observations (Fig. 3a,b). Therefore, on interdecadal timescales, we suggest that more EASM and less NSM precipitation leading to higher EASM/NSM ratios results in lower δ^18^O_s_ values during the cold phases of ENSO/PDO, and vice versa (Fig. 3b).

The δ^18^O_s_ values gradually increase during the late 20th century reaching a maximum of the entire 200-year record. A similar trend was observed in a tree-ring δ^18^O record from Taiwan^36^ and a tree-ring δ^13^C record from SEC^51^. This trend might be caused by an increased frequency of the central Pacific El Niño (CP El Niño) under continued anthropogenic climate warming during recent decades^36,52^. The anomalous low-level anticyclone and Walker circulation associated with CP El Niño events contributes to anomalously dry conditions over southeastern Asia and an intensified winter monsoon with a deeper East Asian trough at 500 hPa resulting in lower temperatures in South China^53^. CP El Niño events can also lead to decreased spring precipitation in South China and increased spring precipitation in the lower reaches of Yangtze River^13^. More NSM precipitation might also contribute to increased δ^18^O_s_ values. We suggest that the highest δ^18^O_s_ values during the late 20th century result from dry conditions in summer, consistent with the results based on tree-ring δ^13^C data from SEC^51^. The synchronously increased δ^13^C values in the EM1 and YQ-15 stalagmites (Supplementary Fig. S5) probably indicate degraded vegetation due to these dry conditions^54^, further supporting our conclusion.

## Methods

### ^230^Th dating

Four subsamples (50–100 mg), drilled using a hand-held carbide dental drill, were analyzed at the Institute of Global Environmental Change, Xi’an Jiaotong University. The chemical procedure used to separate uranium and thorium was similar to that described in Edwards *et al*.^55^. Uranium and thorium isotopes were measured using a multicollector-inductively coupled plasma-mass spectrometer (MC-ICP-MS). Details about the instrumental setup are explained in Cheng *et al*.^56^.

### Stable isotope measurements

For stable isotope analyses (δ^18^O and δ^13^C), a total of 780 subsamples were micro-milled along the central axis of stalagmite EM1. 370 subsamples were micro-milled at a spatial resolution of 0.1 mm from 0 to 13 mm, and 0.05 mm from 13 to 25 mm distance from the top. These samples were measured using a MAT253 isotope ratio mass spectrometer equipped with a MultiPrep system at Xi’an Jiaotong University. Analytical precision of the δ^18^O and δ^13^C analyses was better than 0.06‰ and 0.03‰ (1σ), respectively. 410 subsamples were micromilled along the right side of central axis of stalagmite EM1 at a resolution of 0.07 mm from 25 to 53.7 mm distance from the top, and measured on a Thermo Fisher Delta^plus^ XL isotope ratio mass spectrometer linked to a Gasbench II at the University of Innsbruck. Analytical precision was 0.08‰ and 0.06‰ for δ^18^O and δ^13^C, respectively (1σ)^57^. All isotopic values are reported in the δ notation relative to the Vienna Pee Dee Belemnite (VPDB) standards.

### Layer counting and δ^18^O cycles counting

On the polished profile of stalagmite EM1, visible laminae consisting of a translucent, dense sub-layer (TDSL) and a white, porous sub-layer (WPSL) can be observed (Supplementary Fig. S1). EM1 was imaged using a confocal laser fluorescent microscope (CLFM) at the State Key Laboratory for Manufacturing Systems Engineering, Xi’an Jiaotong University. The visible WPSL is brightly fluorescent while the visible TDSL is non-fluorescent (Supplementary Fig. S1). We identified 200 ± 3 fluorescent laminae between 0 and 53.6 mm from the top of EM1. The 3-year error is a cumulative error from top to bottom. Because the mean growth rate of EM1 between 0 and 45.6 mm from the top is 0.29 mm/year, we obtained a seasonally-resolved δ^18^O record in this section. We counted 159 annual δ^18^O cycles between 0 and 45.6 mm from the top of EM1, which is consistent with the layer-counting result using the CLFM.

### Data sources

Monthly instrumental data from the Nanchang and Jiujiang meteorological station during the period 1951–2010 were obtained from the National Climate Center (NCA, http://ncc.cma.gov.cn). Monthly precipitation δ^18^O data (1988–1992) from Changsha stations were obtained from the Global Network for Isotopes in Precipitation (GNIP, http://www.iaea.org/). The Southern Oscillation Index (SOI) was obtained from http://www.bom.gov.au/climate/current/soihtm1.shtml. Positive SOI values represent El Niño events and negative values represent La Niña events. The Pacific Decadal Oscillation (PDO) index, defined as the leading standardized principal component of monthly sea-surface temperature (SST) anomalies in the North Pacific Ocean, was taken from http://www.ncdc.noaa.gov/teleconnections/pdo. The west Pacific subtropical high (WPSH) plays an important role in governing the variability over East China. The WPSH strength (WPSH_s_) index^58^ is used to represent the intensity of WPSH, with stronger WPSH_s_ concurrent with a south-westward extension of WPSH and vice versa.

### Spectral analyses

Were computed using REDFIT software^59^ on the PAST platform^60^. The 90% confidence level is shown in Supplementary Fig. S7.

## Electronic supplementary material

Supplementary Information
