# Supplementary material for: A 200-year annually laminated stalagmite record of precipitation seasonality in southeastern China and its linkages to ENSO and PDO
Source: Sci Rep. 2018 Aug 17;8:12344. doi: 10.1038/s41598-018-30112-6 (PMC6098110; doi:10.1038/s41598-018-30112-6)
Supplement: Supplementary file 1 — Supplementary Information [file 41598_2018_30112_MOESM1_ESM.docx]

Supplementary information for

**A 200-year annually laminated stalagmite record of precipitation seasonality in southeastern China and its linkages to ENSO and PDO**

Haiwei Zhang^1,2,3^, Hai Cheng^1, 4^, Christoph Spötl^3^, Yanjun Cai^2^^,1^, Ashish Sinha^5^, Liangcheng Tan^2,1^, Liang Yi^6^, Hong Yan^2^, Gayatri Kathayat^1^, Youfeng Ning^1^, Xianglei Li^1^, Fan Zhang^1^, Jingyao Zhao^1^, R. Lawrence Edwards^4^

^1^Institute of Global Environmental Change, Xi’an Jiaotong University, Xi’an 710054, China

^2^Institute of Earth Environment, Chinese Academy of Sciences, State Key Laboratory of Loess and Quaternary Geology, Xi’an 710061, China

^3^Institute of Geology, University of Innsbruck, Innsbruck 6020, Austria

^4^Department of Earth Science, University of Minnesota, Minneapolis, Minnesota 55455, USA

^5^Department of Earth Science, California State University Dominguez Hills, Carson, California 907474, USA

^6^State Key Laboratory of Marine Geology, Tongji University, Shanghai 200092, China

Corresponding: [zhanghaiwei@xjtu.edu.cn](mailto:zhanghaiwei@xjtu.edu.cn)

**Supplementary Figures.**


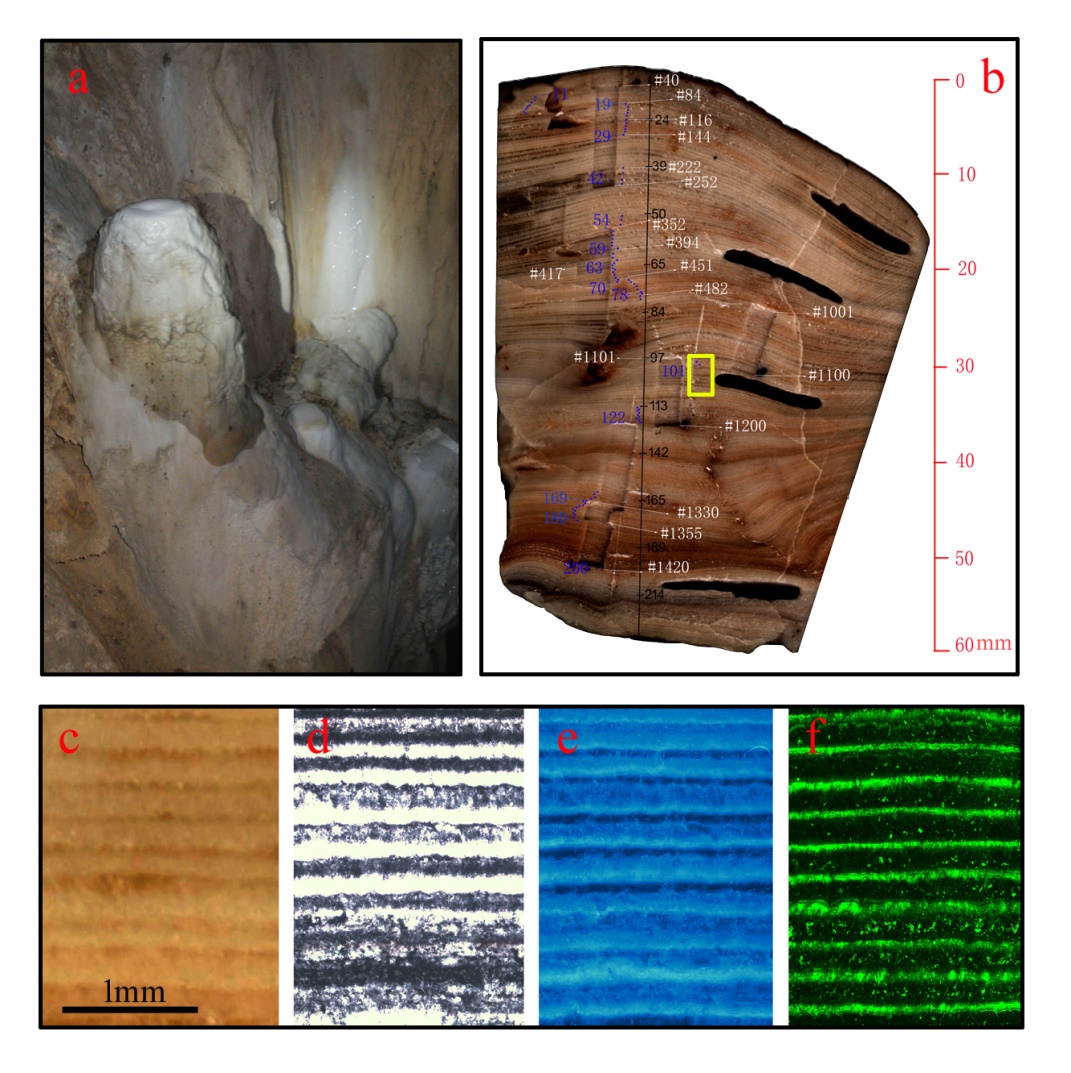


**Supplementary Figure S1.** Information about stalagmite EM1. (a) The stalagmite EM1 in E’mei cave prior to collection in 2009 AD. (b) Longitudinal cross section. The black line shows the scanning track for confocal laser fluorescent microscopy (CLFM). The black numbers from top to bottom indicate the counted layers obtained from CLFM. The white numbers from top to bottom indicate the track number of stable isotope micromilling. The stable isotope tracks corresponding to the counted layers (blue number) were used to build the chronology model. (c-f) From left to right, the image of the yellow rectangle location in (b) was obtained by high-resolution scanning, transmitted-light microscopy, mercury light source UV reflected light and CLFM. The visible white, porous sub-layers are opaque under the transmitted-light microscope, but brightly luminescent under mercury light source UV reflected light and CLFM. The visible translucent, dense sub-layers are transparent under the transmitted-light microscope, but non-luminescent under mercury light source UV reflected light and CLFM.


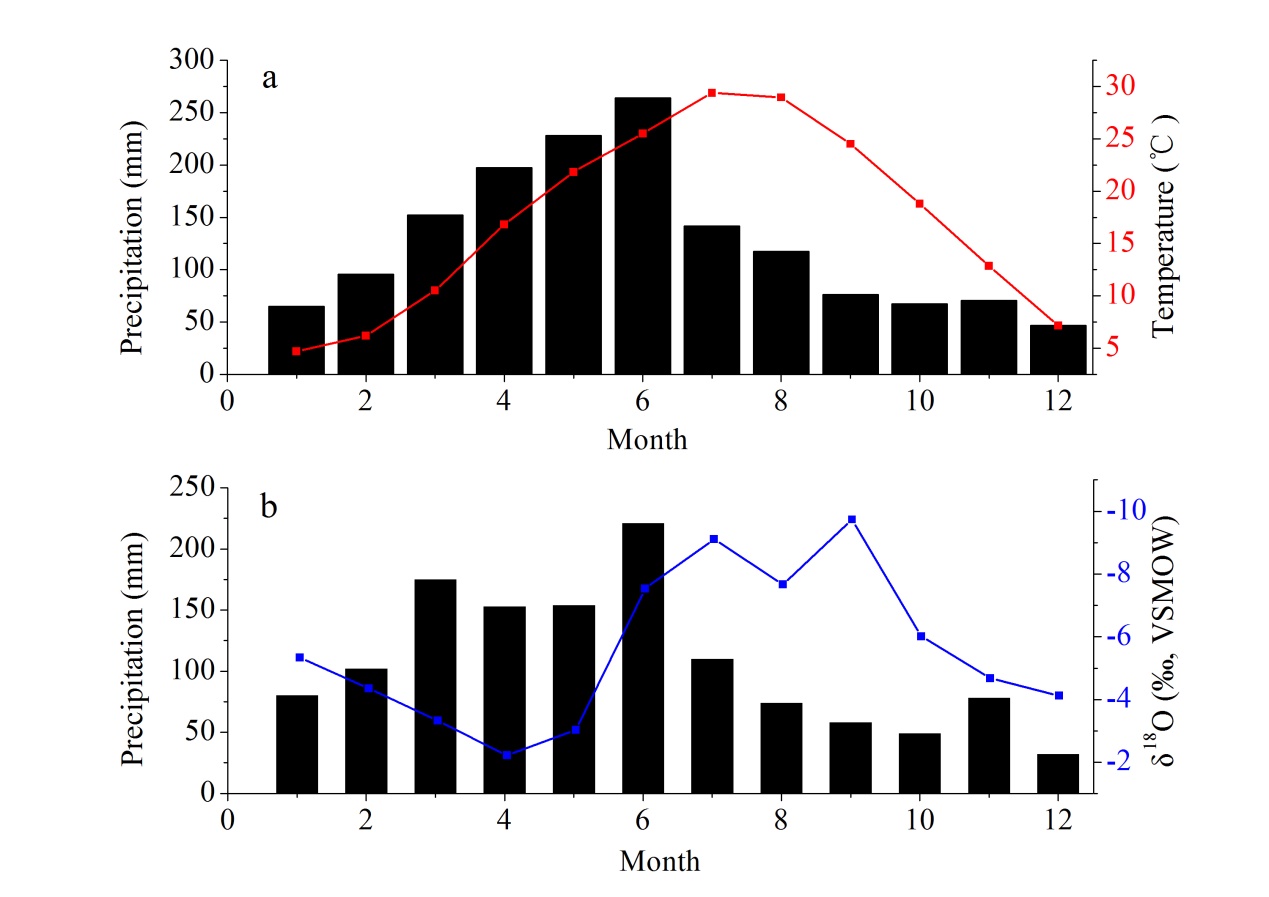


**Supplementary Figure S2.** (a) Mean monthly precipitation amount and temperature during 1951-2010 AD at the Nanchang and Jiujiang stations. (b) Mean monthly precipitation amount and δ^18^O_w_ values during 1988-1992 AD at the nearest GNIP station (Changsha station, about 100 km west of E’mei Cave).


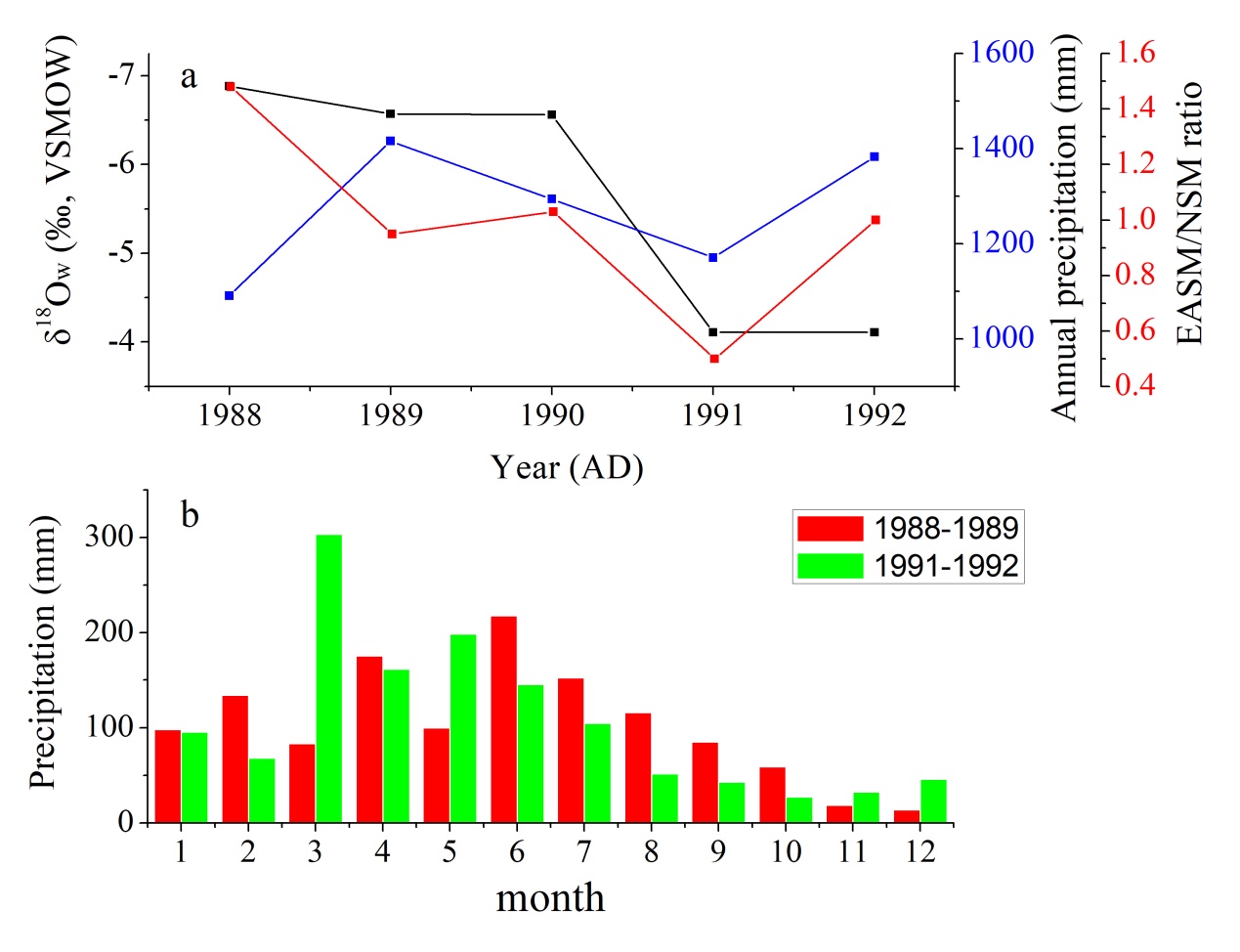


**Supplementary Figure S3.** Comparison among δ^18^O_w_ (black line), annual precipitation (blue line), the ratio of EASM to NSM precipitation amount (red line) between La Niña years (1988-1989 AD) and El Niño years (1991-1992 AD) based on the instrumental datasets from the Changsha GNIP station (ref. S1).


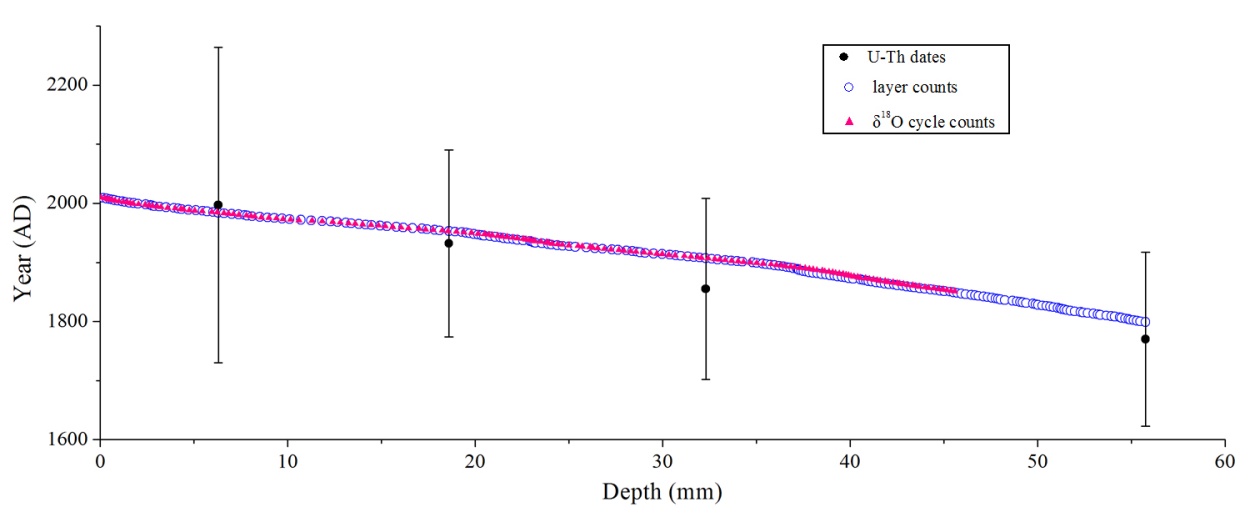


**Supplementary Figure S4.** Age model of stalagmite EM1. The black dots indicate the four ^230^Th dates. The blue circles show the chronology established by lamina counting. The pink triangles show 159 annual δ^18^O_s_ cycles between 0 and 45.6 mm from the top of EM1, consistent with 159 annual laminae.


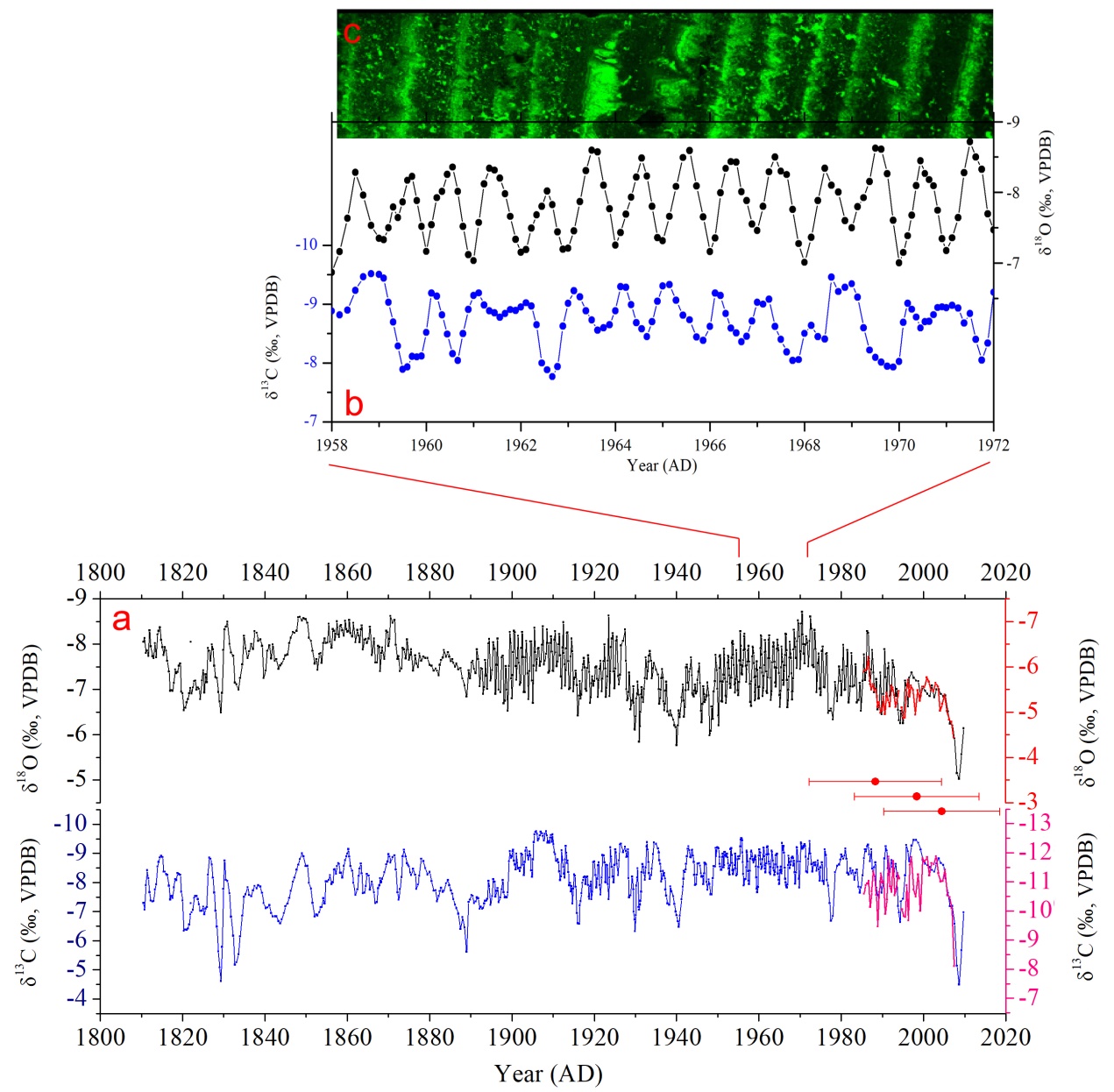


**Supplementary Figure S5**. a) Comparison between δ^18^O (black line) and δ^13^C (blue line) records of stalagmite EM1 and δ^18^O (red line) and δ^13^C (pink line) records of stalagmite YQ15-1. The Chronology of YQ15-1 is based on three ^230^Th ages 2003±14, 1997±15 and 1987±15 AD (with red error bars). The good replication suggests that the chronology of EM1 is accurate and EM1 was deposited under condition close to isotopic equilibrium. b) The clear annual cycles of δ^18^O (black line) and δ^13^C (blue line) in EM1 record consistent with the bright-black couplets under the confocal laser fluorescence microscope (c).


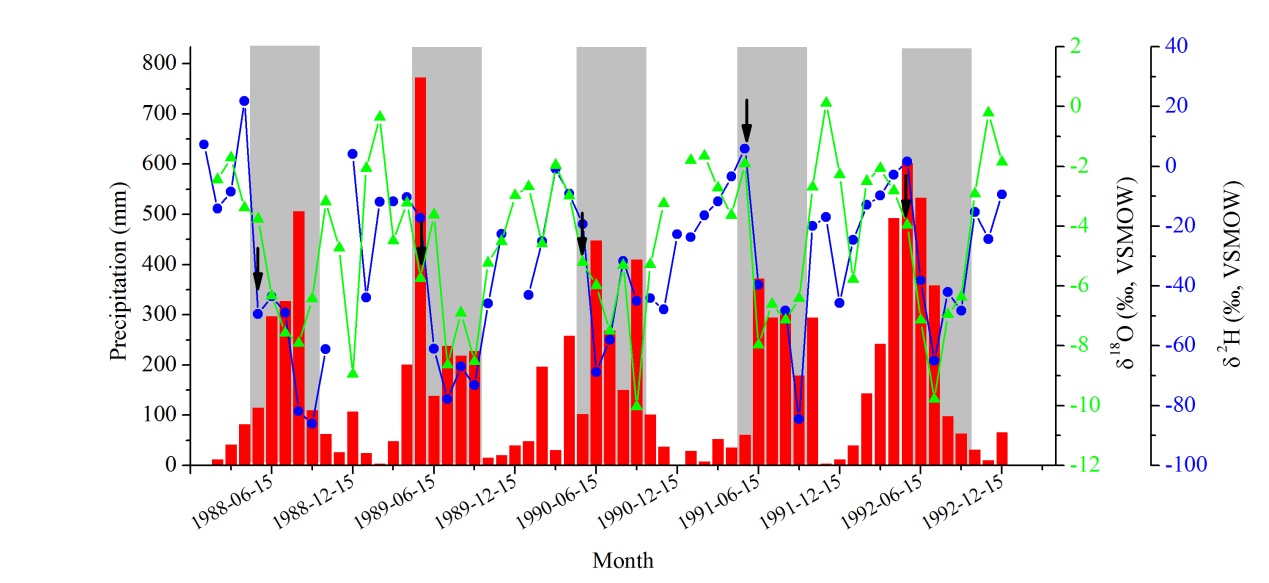


**Supplementary Figure S6.** Precipitation δ^18^O values at the Changsha GNIP station during 1988-1992 AD. The δ^18^O values in May (black arrows) during La Niña years (1988-1989 AD) are more negative than those during El Niño years (1991-1992 AD), indicating that the EASM starts early (late) during La Niña (El Niño) events.


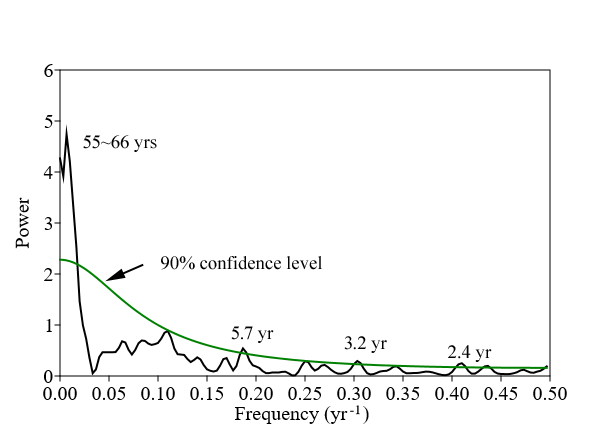


**Supplementary Figure S7.** Power spectral analysis of EM1 δ^18^O record showing significant periods of 2.4, 3.2, 5.7 and 55-66 years. The green line indicates the 90% confidence level.


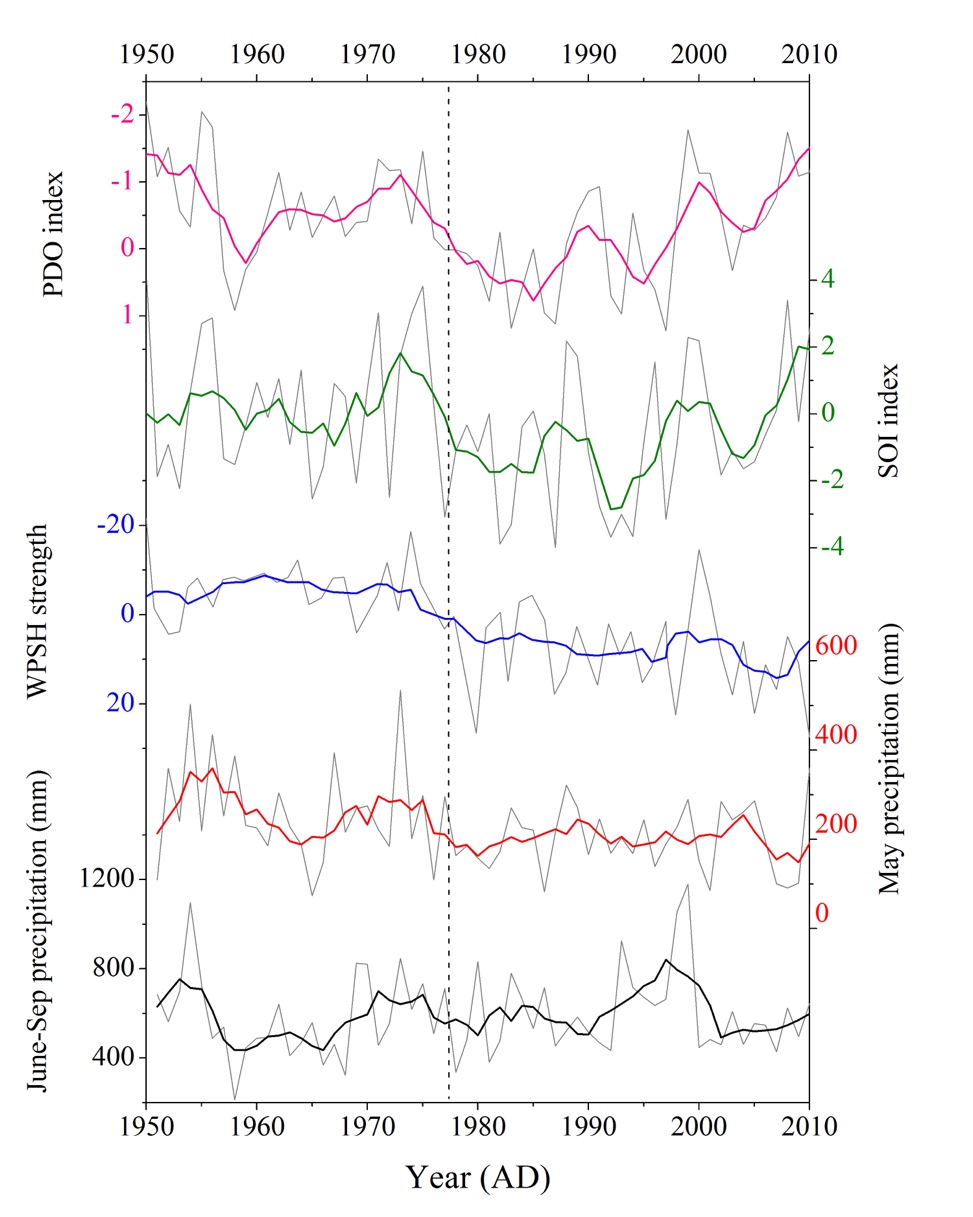


**Supplementary Figure S8.** Comparison between May and June-September precipitation in the study area, PDO, WPSH and SOI index during 1951-2010 AD. The dashed line indicates the ENSO/PDO circulation shift in 1976/1977 AD. There is more May precipitation but less June-September precipitation associated with weakened WPSH strength during the long-term cold phase of PDO/ENSO (1951-1976 AD) than those during the long-term warm phase of PDO/ENSO (1977-2000 AD) associated with a strong WPSH.

**Supplementary Tables.**

**Table S1.** ^230^Th dating results for stalagmite EM1. The error is 2σ.

| Depth | ^238^U | | ^232^Th | | ^230^Th / ^232^Th | | δ^234^U* | | | ^230^Th / ^238^U | | ^230^Th Age | | δ^234^U_Initial_** | | **Age (AD)** | |
| --- | --- | --- | --- | --- | --- | --- | --- | --- | --- | --- | --- | --- | --- | --- | --- | --- | --- |
| mm | (ppb) | | (ppt) | | (atomic x10^-6^) | | | (measured) | | (activity) | | (uncorrected) | | (corrected) | | **(corrected )** | |
| 6 | 236 | ±1 | 3626 | ±119 | 5 | ±0 | 185.7 | | ±2.5 | 0.0043 | ±0.0001 | 396 | ±5 | 186 | ±3 | **1997** | **±267** |
| 18.5 | 289 | ±0 | 2226 | ±58 | 6 | ±2 | 192.9 | | ±2.2 | 0.0030 | ±0.0009 | 271 | ±84 | 193 | ±2 | **1932** | **±158** |
| 32 | 248 | ±0 | 2119 | ±69 | 8 | ±1 | 185.4 | | ±1.5 | 0.0040 | ±0.0004 | 369 | ±36 | 185 | ±2 | **1855** | **±153** |
| 55.5 | 269 | ±1 | 2198 | ±61 | 10 | ±1 | 181.1 | | ±3.3 | 0.0048 | ±0.0004 | 447 | ±36 | 181 | ±3 | **1770** | **±147** |

U decay constants: λ_238_ = 1.55125x10^-10^ (ref. S2) and λ_234_ = 2.82206x10^-6^ (ref. 56). Th decay constant: λ_230_ = 9.1705x10^-6^ (ref. 56). *δ^234^U = ([^234^U/^238^U]_activity_ – 1)x1000. ** δ^234^U_initial_ was calculated based on ^230^Th age (T), i.e., δ^234^U_initial_ = δ^234^U_measured_ x e^λ234xT^. Corrected ^230^Th ages assume the initial ^230^Th/^232^Th atomic ratio of 4.4±2.2 x10^-6^. Those are the values for a material at secular equilibrium, with the bulk earth ^232^Th/^238^U value of 3.8. The errors are arbitrarily assumed to be 50%.

**Table S2.** Oxygen and carbon isotopic data of stalagmite EM1 from E’mei Cave. Ages are determined by counting of annual laminae and δ^18^O cycles. All isotopic values are reported in the δ notation relative to the Vienna Pee Dee Belemnite (VPDB) standards.

Year δ^13^C δ^18^O Year δ^13^C δ^18^O Year δ^13^C δ^18^O

(AD) (‰) (‰) (AD) (‰) (‰) (AD) (‰) (‰)

2009.7 -6.98 -6.14 1952.6 -8.48 -7.71 1900.0 -8.90 -6.80

2009.2 -5.67 -5.56 1952.5 -8.65 -7.74 1899.8 -8.84 -7.24

2008.6 -4.49 -5.03 1952.4 -8.86 -7.59 1899.7 -8.92 -8.02

2008.1 -5.14 -5.15 1952.3 -8.99 -7.16 1899.5 -9.08 -8.27

2007.5 -6.57 -5.92 1952.1 -8.98 -6.83 1899.3 -8.73 -7.51

2007.0 -7.19 -6.20 1952.0 -8.89 -6.70 1899.0 -7.66 -6.53

2006.4 -7.18 -6.24 1951.9 -8.93 -6.86 1898.8 -7.48 -6.99

2005.9 -7.64 -6.51 1951.8 -9.04 -7.23 1898.5 -7.50 -7.51

2005.3 -8.15 -6.87 1951.6 -9.05 -7.76 1898.3 -7.48 -6.75

2004.8 -8.60 -6.85 1951.5 -8.60 -7.90 1898.0 -7.67 -6.69

2004.2 -8.66 -6.90 1951.4 -8.52 -7.32 1897.8 -8.08 -7.74

2003.7 -8.82 -7.00 1951.2 -8.98 -7.17 1897.5 -7.76 -7.70

2003.1 -8.65 -7.00 1951.1 -8.87 -6.73 1897.2 -7.48 -6.94

2002.6 -8.37 -6.82 1950.9 -8.43 -6.74 1896.8 -7.32 -6.99

2002.0 -8.43 -7.08 1950.8 -8.43 -7.17 1896.5 -7.81 -8.12

2001.5 -8.52 -6.85 1950.6 -8.55 -7.75 1896.3 -7.71 -7.54

2000.9 -8.59 -6.90 1950.5 -8.64 -7.72 1896.0 -7.51 -6.90

2000.4 -8.64 -6.97 1950.4 -8.88 -7.46 1895.8 -8.02 -7.89

1999.8 -8.77 -7.06 1950.2 -9.17 -6.21 1895.5 -8.11 -8.04

1999.3 -9.12 -6.86 1950.1 -9.20 -6.42 1895.2 -7.52 -7.42

1998.7 -9.36 -7.18 1949.9 -9.21 -7.01 1894.8 -7.23 -7.19

1998.2 -9.46 -7.19 1949.8 -9.19 -7.42 1894.5 -7.87 -8.11

1997.6 -9.46 -7.25 1949.6 -8.90 -7.58 1894.3 -8.05 -8.01

1997.1 -9.26 -7.37 1949.5 -9.18 -7.76 1894.0 -7.43 -7.01

1996.5 -8.73 -7.26 1949.3 -8.55 -7.18 1893.8 -7.37 -7.30

1996.2 -9.06 -6.63 1949.0 -8.56 -6.95 1893.5 -7.37 -7.65

1995.8 -9.29 -7.09 1948.8 -8.15 -6.35 1893.3 -7.28 -7.05

1995.5 -8.25 -7.19 1948.5 -7.59 -6.62 1893.0 -7.12 -6.90

1995.0 -7.43 -6.25 1948.3 -7.59 -6.08 1892.8 -7.09 -7.45

1994.5 -7.34 -6.53 1948.0 -7.95 -5.98 1892.5 -7.16 -7.47

1994.3 -6.64 -6.25 1947.8 -8.11 -6.86 1892.2 -7.09 -7.07

1994.0 -6.96 -6.32 1947.5 -7.74 -7.15 1891.8 -7.46 -7.67

1993.8 -7.19 -6.54 1947.3 -8.02 -6.86 1891.5 -7.49 -7.88

1993.5 -8.21 -7.01 1947.2 -8.61 -6.82 1891.2 -7.35 -7.36

1993.2 -8.64 -6.85 1947.0 -8.64 -6.77 1890.8 -7.29 -7.45

1992.8 -8.38 -7.49 1946.8 -8.68 -6.72 1890.5 -7.54 -7.63

1992.5 -8.53 -7.64 1946.7 -8.31 -6.89 1890.2 -7.57 -7.59

1992.2 -8.76 -6.96 1946.5 -7.94 -7.07 1889.8 -7.40 -7.24

1991.8 -8.35 -6.86 1946.3 -8.21 -6.68 1889.5 -7.33 -7.42

1991.5 -8.25 -7.58 1946.1 -8.20 -6.27 1889.0 -5.62 -6.84

1991.3 -8.49 -7.46 1945.9 -8.31 -6.52 1888.5 -6.45 -7.08

1991.0 -9.10 -6.86 1945.7 -8.58 -7.14 1888.0 -6.81 -7.24

1990.8 -9.24 -7.27 1945.5 -8.67 -7.57 1887.5 -6.40 -7.47

1990.5 -8.82 -7.89 1945.3 -8.65 -7.39 1887.0 -6.92 -7.44

1990.2 -7.98 -6.90 1945.1 -8.64 -7.21 1886.5 -7.12 -7.57

1989.8 -7.65 -6.45 1944.9 -8.27 -6.95 1886.2 -7.23 -7.50

1989.5 -8.04 -7.37 1944.7 -7.94 -6.91 1885.8 -7.42 -7.58

Continue to next page

**Table S2 (Cont.)**

Year δ^13^C δ^18^O Year δ^13^C δ^18^O Year δ^13^C δ^18^O

(AD) (‰) (‰) (AD) (‰) (‰) (AD) (‰) (‰)

1989.2 -7.49 -6.64 1944.5 -7.91 -7.10 1885.5 -7.61 -7.67

1988.8 -7.57 -6.55 1944.3 -8.25 -6.97 1885.2 -7.72 -7.62

1988.5 -7.82 -7.22 1944.2 -8.70 -6.84 1884.8 -7.90 -7.72

1988.3 -8.68 -7.01 1944.0 -8.91 -6.88 1884.5 -8.08 -7.82

1988.0 -8.52 -6.93 1943.8 -9.13 -6.91 1884.0 -7.97 -7.72

1987.8 -8.35 -7.37 1943.7 -9.11 -7.31 1883.5 -7.72 -7.87

1987.5 -8.47 -7.87 1943.5 -8.86 -7.71 1883.2 -7.67 -7.70

1987.3 -9.14 -7.44 1943.3 -8.87 -7.45 1882.8 -7.81 -7.65

1987.0 -8.73 -7.12 1943.1 -9.10 -7.39 1882.5 -7.70 -7.54

1986.8 -8.96 -7.68 1942.9 -9.24 -6.99 1882.2 -7.53 -7.31

1986.5 -9.30 -8.25 1942.7 -9.16 -7.15 1881.8 -7.35 -7.41

1986.3 -9.24 -8.29 1942.5 -8.90 -7.54 1881.5 -7.73 -7.46

1986.0 -9.07 -7.36 1942.3 -8.64 -7.48 1881.0 -7.71 -7.41

1985.8 -9.14 -7.21 1942.2 -8.44 -6.98 1880.5 -8.04 -7.52

1985.5 -8.83 -7.77 1942.0 -8.36 -6.61 1880.0 -8.36 -7.54

1985.2 -8.24 -7.19 1941.8 -8.40 -6.40 1879.5 -8.27 -7.69

1984.8 -7.83 -6.53 1941.7 -8.12 -6.75 1879.0 -8.49 -7.69

1984.5 -7.65 -7.14 1941.5 -7.85 -7.10 1878.5 -8.74 -7.72

1984.2 -7.85 -6.76 1941.2 -7.42 -6.82 1878.0 -8.81 -7.63

1983.8 -8.43 -6.71 1940.8 -6.86 -6.39 1877.5 -8.23 -7.79

1983.5 -8.33 -7.55 1940.5 -6.48 -6.53 1877.2 -7.92 -7.49

1983.2 -8.10 -7.08 1940.3 -6.67 -6.20 1876.8 -7.78 -7.57

1982.8 -8.40 -7.06 1940.0 -6.87 -5.77 1876.5 -8.27 -7.75

1982.5 -8.31 -7.80 1939.8 -7.00 -6.16 1876.0 -8.46 -7.41

1982.2 -8.49 -7.06 1939.5 -7.25 -6.21 1875.5 -8.26 -7.88

1981.8 -8.27 -6.71 1939.2 -7.53 -6.33 1875.3 -8.34 -7.76

1981.5 -8.33 -7.87 1938.8 -7.70 -6.43 1875.0 -8.55 -7.65

1981.3 -8.81 -7.51 1938.5 -7.87 -6.53 1874.8 -8.60 -7.87

1981.0 -9.16 -6.99 1938.2 -7.24 -6.43 1874.5 -8.66 -8.10

1980.8 -8.92 -6.99 1937.8 -7.28 -6.51 1874.2 -8.88 -7.68

1980.5 -8.81 -7.98 1937.5 -7.55 -7.17 1873.8 -9.12 -7.88

1980.3 -8.89 -7.42 1937.3 -7.75 -6.97 1873.5 -8.89 -7.91

1980.0 -8.96 -6.87 1937.2 -8.13 -6.68 1873.2 -8.18 -7.46

1979.8 -8.79 -7.18 1937.0 -8.32 -6.54 1872.8 -7.77 -7.40

1979.5 -8.62 -7.50 1936.8 -8.25 -6.74 1872.5 -7.40 -7.73

1979.0 -8.57 -6.74 1936.7 -8.39 -7.25 1872.2 -7.66 -7.67

1978.5 -8.34 -7.16 1936.5 -8.12 -7.45 1871.8 -8.44 -7.98

1978.0 -6.85 -6.33 1936.3 -8.03 -7.25 1871.5 -8.93 -8.53

1977.5 -6.68 -6.57 1936.2 -8.38 -6.99 1871.0 -8.86 -8.47

1977.2 -7.28 -6.52 1936.0 -8.64 -6.80 1870.5 -8.68 -8.62

1976.8 -7.89 -6.48 1935.8 -8.76 -6.68 1870.3 -8.89 -8.11

1976.5 -8.08 -7.80 1935.7 -9.03 -7.00 1870.0 -8.97 -7.96

1976.3 -8.94 -7.78 1935.5 -9.17 -7.32 1869.8 -8.72 -8.26

1976.0 -9.14 -7.04 1935.0 -9.33 -7.44 1869.5 -8.62 -8.29

1975.8 -7.95 -7.05 1934.5 -9.37 -7.58 1869.0 -8.34 -7.67

1975.5 -8.05 -7.95 1934.3 -8.97 -7.46 1868.5 -8.13 -7.92

1975.3 -8.68 -7.84 1934.0 -8.47 -7.24 1868.3 -8.00 -7.80

1975.0 -8.51 -6.87 1933.8 -8.25 -7.63 1868.0 -8.14 -7.40

1974.8 -8.06 -7.02 1933.5 -8.12 -7.85 1867.8 -7.90 -7.93

1974.5 -8.45 -7.79 1933.3 -8.31 -7.74 1867.5 -7.80 -7.96

1974.3 -8.75 -7.58 1933.2 -8.69 -7.61 1867.2 -7.32 -7.59

1974.0 -8.40 -6.94 1933.0 -9.12 -7.40 1866.8 -7.29 -7.59

1973.8 -8.70 -7.89 1932.8 -9.39 -7.24 1866.5 -7.66 -8.12

Continue to next page

**Table S2 (Cont.)**

Year δ^13^C δ^18^O Year δ^13^C δ^18^O Year δ^13^C δ^18^O

(AD) (‰) (‰) (AD) (‰) (‰) (AD) (‰) (‰)

1973.5 -8.59 -8.31 1932.7 -9.24 -7.49 1866.0 -8.02 -7.76

1973.3 -8.68 -7.49 1932.5 -8.51 -7.70 1865.5 -7.76 -8.38

1973.0 -8.37 -7.53 1932.3 -8.10 -7.25 1865.2 -8.23 -8.12

1972.8 -8.63 -8.33 1932.1 -8.33 -7.01 1864.8 -8.28 -8.02

1972.5 -8.99 -8.61 1931.9 -8.54 -6.72 1864.5 -7.87 -8.11

1972.3 -9.42 -8.06 1931.7 -8.63 -6.82 1864.2 -7.65 -8.09

1972.0 -8.81 -7.51 1931.5 -8.34 -7.05 1863.8 -7.63 -7.88

1971.8 -8.34 -8.02 1931.4 -7.68 -6.89 1863.5 -8.31 -8.41

1971.5 -8.38 -8.17 1931.2 -7.37 -6.50 1863.3 -8.65 -8.32

1971.4 -8.88 -7.99 1931.1 -7.24 -6.18 1863.0 -8.47 -8.04

1971.2 -9.24 -7.99 1930.9 -7.07 -5.84 1862.8 -8.22 -8.18

1971.1 -9.31 -7.57 1930.8 -7.15 -6.88 1862.5 -7.76 -8.31

1970.9 -9.26 -7.79 1930.5 -8.48 -7.17 1862.0 -7.57 -8.04

1970.8 -9.21 -8.00 1930.4 -8.16 -6.90 1861.5 -7.99 -8.47

1970.6 -9.16 -8.21 1930.2 -7.88 -6.79 1861.2 -8.40 -8.17

1970.5 -9.21 -8.71 1930.1 -6.88 -6.40 1860.8 -8.56 -8.24

1970.3 -9.33 -8.45 1929.9 -6.32 -6.11 1860.5 -8.89 -8.53

1970.2 -9.38 -7.84 1929.8 -6.72 -6.12 1860.3 -9.14 -8.28

1970.0 -8.71 -7.45 1929.6 -6.99 -6.70 1860.0 -8.99 -8.27

1969.8 -7.83 -8.16 1929.5 -7.76 -7.07 1859.8 -8.89 -8.33

1969.7 -8.12 -8.45 1929.2 -8.19 -6.75 1859.5 -8.76 -8.51

1969.5 -8.72 -8.52 1928.8 -7.55 -6.56 1859.2 -8.57 -8.18

1969.3 -9.20 -8.06 1928.5 -7.49 -7.37 1858.8 -8.17 -8.30

1969.2 -9.20 -7.47 1928.3 -8.15 -6.94 1858.5 -8.33 -8.42

1969.0 -8.34 -7.69 1928.2 -9.13 -6.79 1858.3 -8.33 -8.39

1968.8 -7.95 -7.93 1928.0 -8.88 -6.76 1858.0 -8.64 -8.16

1968.7 -8.35 -8.12 1927.8 -8.87 -7.00 1857.8 -8.66 -8.21

1968.5 -8.74 -8.32 1927.7 -9.08 -7.63 1857.5 -8.56 -8.50

1968.4 -8.58 -7.88 1927.5 -9.23 -8.32 1857.3 -8.31 -8.22

1968.3 -8.83 -7.24 1927.0 -9.03 -8.07 1857.0 -8.45 -8.09

1968.2 -8.88 -6.96 1926.5 -9.33 -7.97 1856.8 -8.44 -8.29

1968.1 -8.84 -6.78 1926.3 -9.30 -7.32 1856.5 -8.00 -8.33

1968.0 -8.85 -6.94 1926.0 -8.76 -7.11 1856.2 -7.95 -7.92

1967.9 -8.84 -7.35 1925.8 -8.25 -7.29 1855.8 -8.25 -8.05

1967.8 -8.72 -7.69 1925.5 -8.63 -8.16 1855.5 -8.08 -8.45

1967.7 -8.61 -7.78 1925.3 -9.03 -8.07 1855.2 -8.03 -8.03

1967.6 -8.60 -7.87 1925.1 -9.13 -7.46 1854.8 -8.03 -8.02

1967.5 -8.50 -8.05 1924.9 -8.60 -7.02 1854.5 -7.28 -8.12

1967.4 -8.68 -7.70 1924.7 -8.01 -7.39 1854.2 -7.45 -8.02

1967.3 -8.81 -7.28 1924.5 -8.20 -8.24 1853.8 -7.16 -7.58

1967.2 -8.91 -6.99 1924.3 -8.53 -8.12 1853.5 -7.14 -7.80

1967.1 -8.59 -6.75 1924.1 -8.89 -7.93 1853.2 -6.98 -7.78

1966.9 -7.93 -6.60 1923.9 -9.21 -7.39 1852.8 -6.87 -7.43

1966.8 -7.83 -7.20 1923.7 -8.16 -6.85 1852.5 -6.89 -7.67

1966.7 -7.85 -7.86 1923.5 -7.43 -8.63 1852.1 -6.83 -7.57

1966.6 -7.91 -8.21 1923.3 -7.94 -7.63 1851.6 -7.29 -7.85

1966.5 -8.00 -8.23 1923.2 -8.58 -7.28 1851.2 -7.78 -8.07

1966.4 -8.12 -7.75 1923.0 -8.60 -7.03 1850.7 -7.98 -8.14

1966.3 -8.50 -7.53 1922.8 -8.00 -6.83 1850.3 -8.57 -8.47

1966.1 -9.02 -7.40 1922.7 -8.01 -7.46 1849.9 -8.56 -8.55

1966.0 -9.25 -7.10 1922.5 -8.69 -7.99 1849.4 -8.89 -8.57

1965.9 -9.19 -7.20 1922.3 -8.92 -7.62 1849.0 -9.00 -8.52

1965.8 -9.11 -7.60 1922.0 -8.94 -7.23 1848.5 -8.80 -8.60

Continue to next page

**Table S2 (Cont.)**

Year δ^13^C δ^18^O Year δ^13^C δ^18^O Year δ^13^C δ^18^O

(AD) (‰) (‰) (AD) (‰) (‰) (AD) (‰) (‰)

1965.6 -9.36 -7.77 1921.8 -8.16 -6.96 1848.1 -8.60 -8.59

1965.5 -8.31 -7.94 1921.5 -7.58 -7.63 1847.7 -8.43 -8.29

1965.4 -8.35 -7.48 1921.3 -7.91 -7.45 1847.2 -8.16 -8.25

1965.3 -8.54 -6.96 1921.1 -8.69 -7.19 1846.8 -8.02 -8.27

1965.1 -8.40 -6.61 1920.9 -8.58 -6.83 1846.3 -7.71 -8.14

1965.0 -7.96 -6.87 1920.7 -8.13 -7.23 1845.9 -7.46 -8.02

1964.9 -7.95 -7.36 1920.5 -7.83 -7.50 1845.5 -7.27 -7.77

1964.8 -8.09 -7.85 1920.3 -8.25 -7.48 1845.0 -7.18 -7.75

1964.6 -8.30 -7.98 1920.0 -8.71 -7.18 1844.6 -6.95 -7.67

1964.5 -8.52 -8.10 1919.8 -8.69 -7.08 1844.1 -6.73 -7.57

1964.4 -8.99 -7.89 1919.5 -7.68 -7.28 1843.7 -6.59 -7.53

1964.3 -8.96 -7.48 1919.3 -7.42 -7.13 1843.3 -6.68 -7.45

1964.1 -8.93 -7.06 1919.2 -8.03 -6.82 1842.8 -6.84 -7.67

1964.0 -8.61 -7.15 1919.0 -7.87 -6.70 1842.4 -6.83 -7.63

1963.9 -8.35 -7.48 1918.8 -7.43 -7.38 1841.9 -6.83 -7.99

1963.8 -8.26 -7.61 1918.7 -7.51 -7.59 1841.5 -6.94 -7.68

1963.6 -8.41 -8.03 1918.5 -7.91 -7.75 1841.1 -7.22 -7.97

1963.5 -8.49 -8.04 1918.3 -8.20 -7.47 1840.6 -7.14 -7.85

1963.4 -8.74 -7.94 1918.1 -8.18 -6.97 1840.2 -7.12 -7.41

1963.2 -9.05 -7.59 1917.9 -7.95 -7.01 1839.7 -7.29 -7.25

1963.1 -9.09 -6.95 1917.7 -8.20 -7.48 1839.3 -7.64 -7.96

1962.9 -8.52 -6.76 1917.5 -8.17 -7.69 1838.9 -7.86 -8.10

1962.8 -8.29 -7.25 1917.3 -8.08 -7.55 1838.4 -7.90 -8.07

1962.6 -8.34 -7.69 1917.1 -7.99 -7.42 1838.0 -7.80 -8.25

1962.5 -8.63 -8.19 1916.9 -7.63 -6.88 1837.5 -7.88 -8.28

1962.4 -8.71 -8.09 1916.7 -7.35 -6.79 1837.1 -7.72 -7.89

1962.3 -8.97 -7.68 1916.5 -7.21 -7.18 1836.7 -7.52 -8.00

1962.1 -9.23 -7.26 1916.3 -6.57 -6.86 1836.2 -7.57 -7.84

1962.0 -9.21 -6.92 1916.0 -6.60 -6.42 1835.8 -7.84 -7.69

1961.9 -8.95 -6.96 1915.8 -6.94 -7.09 1835.3 -7.59 -7.90

1961.8 -8.65 -7.39 1915.5 -7.85 -7.41 1834.9 -7.50 -8.18

1961.6 -8.35 -7.83 1915.3 -8.23 -7.26 1834.5 -6.86 -7.55

1961.5 -8.48 -8.09 1915.1 -8.27 -7.22 1834.0 -6.15 -7.25

1961.4 -8.59 -7.82 1914.9 -7.78 -6.96 1833.6 -5.53 -6.99

1961.3 -8.89 -7.53 1914.7 -7.28 -7.84 1833.1 -5.26 -7.11

1961.2 -9.19 -7.29 1914.5 -7.87 -8.12 1832.7 -5.17 -7.15

1961.1 -9.20 -7.03 1914.3 -7.69 -7.72 1832.3 -6.17 -7.74

1960.9 -8.79 -6.85 1914.1 -7.98 -7.03 1831.8 -6.82 -7.81

1960.8 -8.55 -7.37 1913.9 -7.91 -7.00 1831.4 -7.63 -8.00

1960.7 -8.50 -7.77 1913.7 -8.35 -7.62 1830.9 -8.05 -8.49

1960.6 -8.46 -8.17 1913.5 -8.75 -8.26 1830.5 -8.09 -8.38

1960.5 -8.63 -8.19 1913.4 -8.66 -8.24 1830.2 -8.76 -8.32

1960.4 -8.79 -7.91 1913.2 -9.02 -7.90 1829.9 -7.78 -7.87

1960.3 -9.02 -7.47 1913.1 -9.25 -7.42 1829.6 -5.79 -6.92

1960.1 -9.13 -7.05 1912.9 -8.78 -7.08 1829.3 -4.61 -6.49

1960.0 -8.91 -6.81 1912.8 -8.17 -7.55 1829.0 -5.08 -6.73

1959.9 -8.53 -6.80 1912.6 -8.30 -7.80 1828.7 -5.63 -6.94

1959.8 -7.84 -7.04 1912.5 -8.53 -8.48 1828.3 -6.48 -7.25

1959.6 -7.67 -7.42 1912.3 -8.76 -7.92 1828.0 -7.03 -7.27

1959.5 -7.79 -7.62 1912.1 -9.10 -7.02 1827.7 -7.65 -7.52

1959.4 -8.17 -7.45 1911.9 -8.20 -7.28 1827.4 -8.16 -7.88

1959.3 -8.55 -7.28 1911.7 -8.08 -7.75 1827.1 -8.61 -7.99

1959.2 -8.87 -7.09 1911.5 -8.37 -8.22 1826.8 -8.81 -7.63

Continue to next page

**Table S2 (Cont.)**

Year δ^13^C δ^18^O Year δ^13^C δ^18^O Year δ^13^C δ^18^O

(AD) (‰) (‰) (AD) (‰) (‰) (AD) (‰) (‰)

1959.1 -8.92 -6.79 1911.3 -8.69 -7.99 1826.5 -8.85 -7.85

1959.0 -8.85 -6.75 1911.1 -8.79 -7.61 1826.2 -8.27 -7.91

1959.0 -8.79 -6.93 1910.9 -8.74 -7.51 1825.9 -7.16 -7.16

1958.9 -8.81 -7.26 1910.7 -8.74 -7.81 1825.6 -7.10 -7.33

1958.8 -8.74 -7.58 1910.5 -8.97 -8.28 1825.3 -6.90 -6.93

1958.7 -8.68 -7.80 1910.3 -9.03 -8.09 1825.0 -7.17 -7.42

1958.6 -8.75 -7.92 1910.1 -9.31 -7.40 1824.7 -7.03 -7.19

1958.5 -8.79 -7.94 1909.9 -9.65 -7.41 1824.3 -7.02 -6.78

1958.4 -8.89 -7.72 1909.7 -9.39 -7.83 1824.0 -7.25 -7.01

1958.2 -9.09 -7.17 1909.5 -9.62 -8.17 1823.7 -7.43 -7.04

1958.1 -9.05 -6.63 1909.3 -9.54 -8.02 1823.4 -7.53 -6.99

1957.9 -8.81 -6.72 1909.1 -9.36 -7.40 1823.1 -7.53 -7.19

1957.8 -8.40 -7.12 1908.9 -9.42 -7.37 1822.8 -7.46 -7.18

1957.6 -7.95 -7.61 1908.7 -9.37 -8.02 1822.5 -7.06 -7.04

1957.5 -8.06 -7.96 1908.5 -9.62 -8.32 1822.2 -6.98 -6.91

1957.4 -8.39 -7.85 1908.3 -9.76 -7.78 1821.9 -6.90 -7.03

1957.3 -8.72 -7.61 1908.0 -9.67 -7.63 1821.6 -6.55 -6.91

1957.1 -9.04 -7.52 1907.8 -9.44 -7.55 1821.3 -6.42 -6.78

1957.0 -9.09 -7.44 1907.5 -9.60 -8.39 1821.0 -6.37 -6.71

1956.9 -8.52 -7.16 1907.3 -9.69 -7.98 1820.7 -6.38 -6.59

1956.8 -8.12 -7.52 1907.0 -9.74 -7.61 1820.3 -6.34 -6.54

1956.6 -8.11 -7.88 1906.8 -9.61 -7.52 1820.0 -7.37 -6.91

1956.5 -8.11 -8.23 1906.5 -9.49 -7.91 1819.7 -7.68 -6.95

1956.4 -7.93 -8.17 1906.3 -9.43 -7.89 1819.4 -8.27 -7.57

1956.3 -7.89 -7.87 1906.1 -9.66 -7.66 1819.1 -8.29 -7.52

1956.2 -8.29 -7.64 1905.9 -9.76 -7.41 1818.8 -8.32 -7.38

1956.1 -8.70 -7.51 1905.7 -9.73 -7.82 1818.5 -8.08 -7.36

1956.0 -9.03 -7.50 1905.5 -9.65 -8.36 1818.2 -8.11 -7.44

1955.9 -9.44 -7.33 1905.3 -9.38 -7.52 1817.9 -7.83 -7.14

1955.8 -9.50 -7.35 1905.1 -8.79 -6.70 1817.6 -7.46 -6.97

1955.7 -9.52 -7.53 1904.9 -8.19 -7.19 1817.3 -7.53 -6.98

1955.6 -9.47 -7.96 1904.7 -8.41 -8.06 1817.0 -7.41 -6.97

1955.5 -9.23 -8.28 1904.5 -8.92 -8.06 1816.7 -7.89 -7.39

1955.3 -8.90 -7.63 1904.3 -9.07 -7.40 1816.3 -8.26 -7.63

1955.2 -8.82 -7.16 1904.0 -8.78 -6.78 1816.0 -8.29 -7.85

1955.0 -8.89 -6.87 1903.8 -8.92 -7.35 1815.7 -8.37 -7.93

1954.8 -8.79 -6.98 1903.5 -9.20 -8.07 1815.4 -8.37 -7.76

1954.7 -8.28 -7.27 1903.3 -9.09 -7.70 1815.1 -8.87 -7.93

1954.5 -8.24 -7.86 1903.1 -8.70 -6.94 1814.8 -8.91 -8.09

1954.4 -8.46 -7.22 1902.9 -8.25 -6.94 1814.5 -8.86 -8.37

1954.3 -8.99 -7.14 1902.7 -8.61 -7.71 1814.2 -8.70 -8.29

1954.2 -8.98 -6.93 1902.5 -8.94 -8.27 1813.9 -8.54 -8.21

1954.1 -8.59 -7.11 1902.3 -8.99 -7.91 1813.6 -7.93 -7.71

1953.9 -8.43 -7.53 1902.1 -8.50 -7.15 1813.3 -7.75 -8.02

1953.8 -8.36 -7.77 1901.9 -8.38 -7.10 1813.0 -7.36 -7.69

1953.7 -8.29 -8.00 1901.7 -8.50 -7.58 1812.7 -7.35 -7.73

1953.6 -8.42 -8.06 1901.5 -8.48 -8.18 1812.3 -7.38 -7.95

1953.5 -8.67 -8.02 1901.3 -8.66 -7.68 1812.0 -7.67 -8.30

1953.4 -8.94 -7.69 1901.1 -8.99 -7.21 1811.7 -8.15 -7.74

1953.3 -9.18 -7.25 1900.9 -8.44 -7.51 1811.4 -8.42 -7.86

1953.1 -9.14 -7.03 1900.7 -8.61 -8.26 1811.1 -7.90 -7.80

1953.0 -8.58 -7.12 1900.5 -8.74 -8.29 1810.8 -7.06 -8.13

1952.9 -8.26 -7.31 1900.3 -9.13 -7.82 1810.5 -7.29 -8.05

1952.8 -8.31 -7.46 1900.2 -9.06 -7.31

**Reference**

S1. Zhang, H.W. *et al.* Precipitation seasonality in the area of spring persistent rain in southeastern China and its palaeoclimatic implication. *Clim. Dyn.* Under review.

S2. Jaffey, A.H. *et al.* Precision measurement of half-lives and specific activities of ^235^U and ^238^U. *Phys. Rev.* C**4**, 1889-1906 (1971).
